# Cx47 Phosphorylation Exacerbates White Matter Damage and Kainic Acid Induced Epilepsy

**DOI:** 10.1111/cns.70672

**Published:** 2025-11-26

**Authors:** Yi Li, Haohan Lin, Jiayu Liu, Jie Chen, Kaifeng Shen, Ningning Chen, Songyang Xiang, Duan Wang, Nong Xiao, Tingsong Li

**Affiliations:** ^1^ Department of Rehabilitation Children's Hospital of Chongqing Medical University National Clinical Research Center for Child Health and Disorders, Ministry of Education Key Laboratory of Child Development and Disorders Chongqing China; ^2^ Chongqing Key Laboratory of Child Neurodevelopment and Cognitive Disorders Chongqing China; ^3^ Department of Neurology, Chongqing Emergency Medical Center Chongqing University Central Hospital Chongqing China; ^4^ Department of Neurosurgery, Xinqiao Hospital Third Military Medical University (Army Medical University) Chongqing China

**Keywords:** CaMKII, Cx47, epilepsy, oligodendrocyte, phosphorylation

## Abstract

**Aims:**

Growing evidence implicates dysfunctional myelin in the pathogenesis of temporal lobe epilepsy (TLE). Connexin 47 (Cx47), an oligodendrocytic gap junction protein, maintains myelin integrity. This study investigates the role of Cx47 in myelin impairment and seizure progression in TLE.

**Methods:**

Cx47 and phosphorylated Cx47 (p‐Cx47) expression was analyzed in human and mouse TLE brain tissues via Western blot and immunofluorescence. Candidate Cx47 phosphorylation kinases revealed by single‐cell RNA sequencing were validated through immunofluorescence, protein docking, and co‐immunoprecipitation. TLE mice were treated with the CaMKII inhibitor KN93 to evaluate its effects on demyelination and seizure burden.

**Results:**

In a experimental mouse model, phosphorylated Cx47 (p‐Cx47) was significantly upregulated, recapitulating a similar trend observed in human TLE tissues. This upregulation was accompanied by marked demyelination in the TLE animals. In mice, increased levels of Cx47 and p‐Cx47 were associated with elevated CaMKII and phosphorylated CaMKII (p‐CaMKII). The interaction between Cx47 and CaMKII was further confirmed. Moreover, administration of KN93 suppressed the upregulation of Cx47 and p‐Cx47, thereby mitigating demyelination and reducing seizure progression.

**Conclusions:**

CaMKII‐mediated Cx47 expression and phosphorylation promote demyelination and seizure progression in TLE. Targeting Cx47 phosphorylation may offer a therapeutic strategy for TLE.

## Graphical abstract

The upregulation of phosphorylated Cx47 (p‐Cx47), driven by CaM/CaMK‐dependent phosphorylation, plays a critical role in seizure progression in TLE. The CaMKII inhibitor KN93 suppressed Cx47, thereby reducing its phosphorylation, alleviating demyelination, and attenuating seizure progression. Therefore, targeting Cx47 phosphorylation represents a promising therapeutic strategy.

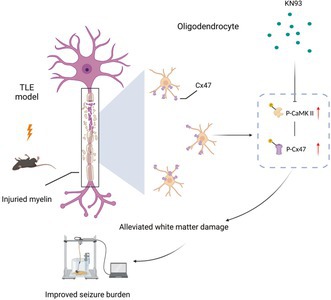

## 1 Introduction

Epilepsy is a prevalent chronic neurological disorder, affecting over 70 million individuals globally [1, 2]. This condition imposes a significant burden on healthcare systems, individuals, and their families [3, 4, 5]. Temporal lobe epilepsy (TLE), which constitutes approximately one‐third of all epilepsy cases, is the most common form of drug‐resistant epilepsy [6, 7].

Beyond neurons, glial dysfunction is a well‐established contributor to the pathogenesis of TLE. Reactive astrocytes disrupt glutamate and potassium homeostasis, promote neuroinflammation, and alter the synaptic environment, thereby driving hyperexcitability [8]. Meanwhile, emerging evidence suggests that oligodendrocytes (OL) are associated with seizure exacerbation in epilepsy, as adaptive myelination is hypothesized to enhance neural network synchrony [9, 10]. Abnormalities in white matter microstructure have been documented in both human epilepsies and rodent models of various epilepsy forms [11, 12, 13]. Notably, acute encephalitis mediated by anti‐myelin oligodendrocyte glycoprotein (MOG) antibodies is a common neurological demyelinating disease in clinical practice, with 21.3% of patients experiencing seizures [14]. Given that myelinating OL facilitate the rapid propagation of action potentials along ensheathed axons, they represent a potential target for seizure control.

Cx47, a gap junction protein predominantly expressed on OL somata and along myelinated fibers [15], has been implicated in demyelinating diseases by regulating neuroinflammation [16] and disrupting gap junctions with other connexins [17]. The gap junctions formed between astrocytes (AST) and OL in the brain primarily result from the docking of Cx43 on AST end feet with Cx47 on OL bodies [18, 19]. Astrocytic–oligodendroglial interactions are increasingly recognized as important regulators of myelin health [20]. In knockout mice, both Cx43 and Cx47 have been shown to play an essential role in maintaining the structural and functional integrity of myelin [21]. Additionally, reduced expression of Cx43 and Cx47 has been observed in multiple sclerosis, a condition characterized by central demyelination [22], indicating that OL survival and function depend on the normal functioning of Cx43/Cx47 gap junctions.

Despite these findings, data on the role of Cx47 in the progression of TLE remain limited. To address this gap, the present study quantitatively assessed the expression of Cx47 and its phosphorylation status in human TLE specimens and animal models, as well as the potential impact of phosphorylated Cx47 on seizure progression.

## 2 Materials and Methods

### 2.1 Human Specimens

All patients with TLE were re‐diagnosed based on histopathological diagnosis after surgery. Specimens for the control group were obtained from the temporal lobes of surgical donors without seizures or epilepsy. After surgical removal, specimens were preserved in a cryogenic refrigerator at −80°C quickly until further use. A total of six TLE and six control group specimens (each comprising 4 males and 2 females) were obtained from Xinqiao Hospital, Army Medical University. The control specimens were assigned to two subgroups according to the primary pathological diagnosis: Con‐1 (tumor‐related pathologies, including meningioma, glioblastoma, *n* = 1 each, and cyst, *n* = 1; total *n* = 3) and Con‐2 (hemorrhagic diseases, including subdural hematoma, subarachnoid hemorrhage, and basal ganglia hemorrhage, *n* = 1 each; total *n* = 3). Detailed clinical information and subgroups of the patients are provided in Table S1. Ethics approval was obtained from Army Medical University Committee (approval number: 2022‐361‐01). The prefixed temporal specimens of humans were cut into 60‐μm‐thick sections for immunofluorescence staining [23].

### 2.2 Animal Model of TLE

Eight‐week‐old male C57BL/6J mice were used for all the experiments. Mice were housed under constant temperature (25°C ± 1°C) and humidity (50%) conditions with a 12 h light/dark cycle and were provided with food and water ad libitum. All experiments involving animals followed protocols in accordance with the guidelines established by the Animal Ethics Committee of Chongqing Medical University for the correct use of laboratory animals in research. All efforts were made to minimize the number of subjects and their suffering.

TLE mouse model was induced by kainic acid (KA) as described in previous research [24]. Briefly, the mice were injected with KA (30 mg/kg) intraperitoneally to induce status epilepticus (SE). Seizures were classified based on the Racine scale [25] as follows: “Mouth and facial movements” (level I), “head nodding” (level II), “forelimb clonus” (level III), “seizures with rearing” (level IV), and “seizures with rearing and falling” (level V). Once reaching a seizure state over level IV and lasting for 30 min, diazepam (0.5 mg/kg) was injected intraperitoneally to terminate SE. The control group received only 0.9% sodium chloride solution and diazepam at the corresponding time points. Mice that failed to develop SE or died were excluded. Mice exhibiting spontaneous seizures 4 weeks after KA administration were regarded as TLE mice. Removal of dead and unsuccessful mice resulted in an overall modeling pass rate of 85% of the TLE model process.

### 2.3 Chemicals and Reagents

**Table:** 

Product	Company	Catalogue no.	Application
Kainic acid	Cayman	58002	30 mg/kg
Membrane protein extraction kit	Abcam	ab65400	—
Whole protein extraction kit	Keygen Biotec	KGB5303	—
Rabbit anti‐Cx47	Thermo Fisher	36‐4700	IF 1:1000, WB 1:2000
Chicken anti‐GFAP	Abcam	ab279290	IF 1:5000
Rabbit anti‐CaMKII	Abcam	ab92332	WB 1:10,000
Rabbit anti‐CaMKII (phospho T286)	Abcam	ab124880	WB 1:4000
Rabbit anti‐calmodulin (CaM) 1/2/3	ABclonal	A4885	IF 1:1000
Rabbit anti‐E‐cadherin (loading control)	ABclonal	A20798	WB 1:1000
Rabbit anti‐GAPDH (loading control)	Proteintech	60004‐1‐Ig	WB 1:5000
Rabbit anti‐β‐actin (loading control)	ABclonal	AC026	WB 1:100,000
KN93	MedChemExpress	HY‐15465	0.1 mg/kg/day*7 days
Rabbit anti‐CaMKII	MedChemExpress	HY‐P80445	IP 1:100
Mouse anti‐Cx47	Thermo Fisher	4A11A2	IP 1:100/IF 1:200
Immunoprecipitation Kit with protein A + G magnetic bead	Beyotime	P2179S	—
Protein A + G magnetic bead	MedChemExpress	HY‐K0202	—
Rabbit anti‐CaMKII	HUABIO	ET1608‐47	IF 1:300
Guinea pig anti‐Olig2	OasisBiofarm	OB‐PGP040	IF 1:500

### 2.4 Immunofluorescence (IF) Staining

After permeabilizing and blocking, the coronal frozen sections were incubated with mixed primary antibodies Cx47/GFAP overnight at 4°C, followed by the application of suitable secondary antibodies staining in the dark. Images were captured using a confocal microscope at CA1, CA3, and dentate gyrus (DG) area. The fluorescence signals in GFAP (green) and Cx47 (red) channels were selected as ROI, and then computed the colocalization area, which was the actual functional Cx47 [26]. As the previously reports [23], the number of plaques, plaque size, and mean fluorescence intensity of colocalization signals were counted. For human sections, images were captured using Z‐stack of 10 μm (each step of 0.85 μm) and processed with deconvolution. For the assessment of CaMKII and Cx47 colocalization, consistent with previous description, primary antibodies against CaMKII, Olig2, and Cx47 were employed for imaging. Confocal microscopy was utilized to capture images specifically from the CA3 region. The CaMKII (green) and Cx47 (red) channels were subsequently selected for line‐scan analysis.

After capturing, all image processing was performed using the imaging software program NIS‐elements AR (Nikon) and Image J software (NIH).

### 2.5 Western Blotting (WB)

After euthanasia, rapidly extract the mouse brain and immediately immerse it in ice‐cold PBS. Next, transfer the brain to a dissection dish containing ice‐cold PBS or place it on an ice‐cold surface. Under a dissecting microscope, carefully identify the corpus callosum, a prominent white fiber bundle situated between the cerebral hemispheres. Using fine scissors or scalpel blades, meticulously dissect along its anatomical borders. Rinse the isolated corpus callosum with fresh ice‐cold PBS for immediate use or store at a cryogenic refrigerator at −80°C.

Membrane and whole protein were obtained from the hippocampal and corpus callosum tissue separately using a protein extraction kit. Following the quantification of protein concentration using the bicinchoninic acid method, the protein was separated by 10% polyacrylamide gel electrophoresis and transferred onto polyvinylidene fluoride (PVDF) membranes. The membrane was blocked for 1 h at room temperature in phosphate buffered saline containing 5% bovine serum albumin and further incubated with specific primary detection antibodies overnight at 4°C. Subsequently, peroxidase‐conjugated secondary antibodies were applied to the same membranes for another 1 h at room temperature. Glyceraldehyde‐phosphate dehydrogenase (GAPDH) and β‐actin were used as whole protein internal references. E‐cadherin was used as membrane protein internal reference. Protein bands were visualized using clarity western electro‐chemiluminescence substrate (Bio‐Rad) and analyzed by densitometry using ImageJ software (NIH).

### 2.6 **Single‐cell RNA sequencing (**scRNA‐Seq) and Bioinformatics Analysis

To systemically explore the molecular mechanism underlying epilepsy phenotypes, we performed scRNA‐Seq on mixed samples of bilateral hippocampal tissues from three adult mice (after 8 weeks injection with KA) and from three wild‐type controls (after 8 weeks injection with 0.9% sodium chloride solution).

After dissociation of entire hippocampus samples were mechanically and enzymatically dissociated, single cells were sorted using microfluidic technology. Freshly prepared single‐cell suspensions were adjusted to cell concentration between 700 and 1200 cells/μL according to 10 × Genomics Chromium Next GEM Single Cell 3′ Reagent Kits v3.1 (no. 1000268). The constructed libraries were subjected to high‐throughput sequencing using the Illumina Nova 6000 PE150 platform. ScRNA‐seq service was provided by OE Biotech (Shanghai, China). Then, we used the mouse reference genome (GRCm38) for read alignment, barcode assignment, and unique molecular identifier (UMI) counting.

After initial quality control of sequencing results, data were filtered and normalized using the Seurat R package. The data were first filtered, and the parameters were set as nFeature > 250, nCount > 1000, precent.mt < 25%. Cells with detectable expression of fewer than 250 genes or fewer than 1000 UMI counts or greater than 25% UMIs from mitochondrial gene transcripts (measure of cell viability) were removed from further analysis. Method = “LogNormalize,”, scaling factor = 10,000 to normalize. Batch effects were removed by the fastMNN function of the Scran package, with the input of the normalized counts from individual datasets. The top 20 principal components were clustered by the FindClusters function with a resolution of 0.5. The clustering results were reduced in dimension and visualized using the UMAP algorithm. The marker genes of each cell subpopulation were screened according to the information of Cell Marker2.0 database and the cell population was annotated with reference to the recent high‐throughput sequencing results of mouse hippocampus or mouse cortex [27, 28]. First, the expression distribution of *Gjc2* (Cx47) in the two groups of OL subsets was checked, and then the FindAllMarkers function was run to find out the differentially expressed genes (DEGs) in *Gjc2* positive OL cells. DEGs selection criteria: significant fold changes (adjusted *p* < 0.05), average log_2_ fold change > 1. Gene Ontology (GO) and Kyoto Encyclopedia of Genes and Genomes (KEGG) enrichment analysis was performed on the upregulated genes, and the GO and KEGG analysis was performed using KOBAS database [29]. Combined with GO results, the genes related to *Gjc2* were searched in GeneMANIA database, and the expression of interested genes was drawn by heatmap. The volcano plot and bar plot were drawn using ggplot2 package. The violin plot was generated by VlnPlot and the *t*‐test was performed for the upregulated or downregulated genes.

### 2.7 Oligodendrocyte Lineage Functional Scoring

Three custom gene sets were defined based on previously reported [30]: Mature Oligodendrocyte (MOL) signature gene set: *Klk6*, *Apod*, *Slc5a11*, *Pde1a*; Myelin‐forming Oligodendrocyte (MFOL) signature gene set: *Mal*, *Mog*, *Plp1*, *Opalin*, *Serinc5*; Newly formed Oligodendrocyte (NFOL) signature gene set: *Tcf7l2*, *Casr*, *Cemip2*, *Itpr2*. Cells were scored for these gene sets using the AddModuleScore function from the Seurat R package. Subsequently, the average score for each cluster was calculated based on the clustering results, and a heatmap was generated using the pheatmap R package.

### 2.8 Mitochondrial Function Scoring and Calcium‐Related Signaling Pathway Scoring

For mitochondrial function scoring, the following gene sets/pathways were utilized: “GOBP_OXIDATIVE_PHOSPHORYLATION”, “REACTOME_CITRIC_ACID_CYCLE_TCA_CYCLE”, “REACTOME_PYRUVATE_METABOLISM”, and “WP_GLYCOLYSIS_AND_GLUCONEOGENESIS”.

Pathway activity scores for all OL lineage cells were calculated using the AUCell algorithm, and subsequently visualized as UMAP density plots using the Nebulosa package. Furthermore, all mitochondria‐related gene sets were filtered from the Molecular Signatures Database (MSigDB) mouse gene sets v2025.1 using “mitochondria” as a keyword, and Gene Set Enrichment Analysis (GSEA) was performed on the OL subcelltypes of interest. Regarding calcium‐related signaling pathway scoring, three calcium‐response pathways were identified from the Gene Ontology (GO) enrichment results of differentially expressed genes in epileptic OL: “CELLULAR_RESPONSE_TO_CALCIUM_ION”, “NEGATIVE_REGULATION_OF_CALCIUM_ION_TRANSPORT”, and “NEGATIVE_REGULATION_OF_ENDOPLASMIC_RETICULUM_CALCIUM_ION_CONCENTRATION”. The corresponding gene sets were downloaded from the Molecular Signatures Database (MSigDB). Pathway activity scores for all OL lineage cells were calculated using the AUCell algorithm and visualized as UMAP density plots using the Nebulosa package. Additionally, all calcium‐related gene sets were filtered from MSigDB using “calcium” as a keyword, and GSEA was performed on the OL subpopulations of interest.

### 2.9 GSEA Analysis

Differential genes from the subpopulations of interest were identified using the org.Mm.eg.db and GSEABase R packages. Subsequently, Gene Set Enrichment Analysis (GSEA) was performed to enrich the input target gene sets.

### 2.10 Cell Trajectory Inference

Slingshot [31], Monocle3 [32] were used to perform cell trajectory inference. Trajectory inference analyses based on different algorithms were used to corroborate each other. Slingshot computes the pseudotime by proposing a simultaneous principal curves algorithm to construct smooth curves from the minimum spanning tree by the R package slingshot. Monocle3 generates the trajectory using the principal graph algorithm.

### 2.11 Protein Docking Simulation

The structure of human Cx47 monomer (model ID: AF‐Q5T442‐F1‐v4) was retrieved from the AlphaFold Protein Structure Database. A 12‐mer structure for human Cx47 was obtained from SWISS‐MODEL (model ID: 7jmd.1.A), which is based on Sheep Connexin‐46 resolved at 2.5 Å resolution in Lipid Class 1 [33]. The human CaMK2A protein structure (model ID: 5ig3) [34] was obtained from the Protein Data Bank (PDB).

Molecular docking was performed using the HDOCK service [35]. Candidate models were filtered based on docking score, Confidence Score, and root mean square deviation (RMSD) values. A confidence score higher than 200 was considered indicative of an interaction. Specifically, model 1 was selected for the Cx47 12‐mer‐CaMK2A interaction, and model 3 for the Cx47 monomer‐CaMK2A interaction. Interacting amino acid residues between the two proteins were subsequently visualized using PyMOL (version 3.1).

### 2.12 Co‐Immunoprecipitation **(Co‐IP)**

Co‐IP assays were performed using mouse hippocampus tissue and a Beyotime Immunoprecipitation Kit with Protein A + G Magnetic Beads. According to the manufacturer's manual, protein extracted using IP Lysis Buffer was incubated overnight (16 h) at 4°C with primary antibodies: either rabbit anti‐CaMKII, mouse anti‐Cx47, or their respective isotype controls (rabbit IgG or mouse IgG, 1:200). Following this, the antibody–protein complex was incubated with Protein A + G Magnetic Beads at room temperature for 2 h. After several washes, the immunoprecipitated protein complex was eluted from the beads and subsequently identified by immunoblotting.

### 2.13 KN93 Administration

KN93 is a widely used CaMKII inhibitor that prevents CaMKII activation by competitively inhibiting Ca^2+^/CaM binding to CaMKII. KN93 was also able to prevent CaMKII activation by autophosphorylation, oxidation, or glycosylation of Thr286. However, it does not affect the function of already activated CaMKII [36].

The mice were divided into four groups according to whether they were TLE mice or whether KN93 administration or not: Con + artificial cerebrospinal fluid (aCSF), Con + KN93, TLE + aCSF, and TLE + KN93. The KN93 intrathecal injection began on the second day after modeling at a dose of 0.1 mg/kg/day and continued for 1 week, and the subsequent experiments were performed 8 weeks after modeling.

### 2.14 Luxol Fast Blue Myelin Stain

In brief, the coronal brain sections were dehydrated in 35% and 70% ethanol for 5 min each before being placed in a 1% Luxol Fast Blue (LFB) solution and incubated overnight at 65°C. The next day the slides were allowed to cool down to room temperature before being rinsed in 95% ethanol, followed by distilled water. The slides were then destained for 30 s in a 0.05% lithium carbonate solution, and then rinsed in 70% ethanol. The slides were placed in fresh 70%, 95%, and 100% ethanol solution for 5 min before being transferred to xylene for 5 min. The slides were then cover slipped for later imaging. Three separate nonoverlapping pictures were taken of both the injured and sham nerves with a Leica DM5000B microscope. FIJI software was used for subsequent image analysis. The thickness of the corpus callosum was measured as the straight distance at the center of the corpus callosum, and the optical density (OD) value of the blue staining signal in the corpus callosum and CA3 region of the hippocampus was measured.

### 2.15 Transmission Electron Microscope

After perfusion with 4% paraformaldehyde, the hippocampus was quickly sorted out on ice, cut into 1 mm^3^ small tissue blocks in electron microscope fixative, and stored in the dark at 4°C. Ultrathin sections at 80 nm were made after fixation with 1% osmic acid, dehydration, infiltration and embedding. The pieces of copper mesh were stained with a 2% uranyl acetate saturated alcohol solution in the dark, dried, and observed under a transmission electron microscope. The images were collected and analyzed at 1500×, 4000×, and 10,000× magnification, respectively. Three regions were randomly selected from each tissue, and five images were collected from each region at low, medium, and high magnification for image analysis using FIJI. The percentage of pathological changes, inner and outer diameter, G‐Ratio and average thickness of the myelin sheath in the hippocampus were measured and calculated.

### 2.16 Seizure Burden Evaluation

Seven weeks after inducing SE, three screw electrodes were inserted bilaterally through the skull over the cortex, as previously described and in agreement with brain atlas coordinates (AP −2.5 mm, ML ±2.0 mm; AP +1.0 mm, ML −1.0 mm). Mice were allowed to recover for a week after surgery. Then each animal was transferred to an observation box and their electrodes were connected to an EEG100C amplifier and video‐electroencephalography (EEG) recording for 2 h, with the EEG signals filtered at 0.1–500 Hz and digitalized at 1 kHz for subsequent analysis. Visual EEG analysis was conducted using Lab Chart 8 Reader. Seizure behaviors in all groups were simultaneously evaluated with the EEG recordings. The EEG raw data were preprocessed using 0.5 Hz high‐pass filtering. Firstly, a 10‐min seizure‐free electroencephalogram was selected as the baseline, and the baseline amplitude level of the mice was measured. The EEG signal with an amplitude higher than two times the baseline that lasts more than 15 s was screened out as an epileptic seizure, and the duration of each seizure was recorded. Spikes were detected using a threshold value of 200 μV to a maximum of 5000 μV. The minimum spike duration was set to 5 ms and the maximum duration to 70 ms. If there were three or more spikes at the same time and the total duration was < 5 s, it was defined as a spike train, and spike trains had a minimum spike interval of 50 ms and a maximum spike interval of 1 s [37].

### 2.17 Statistical Analysis

Statistical analysis was conducted using SPSS 27.0 and GraphPad Prism (v9.2.0). Quantitative data were expressed as mean ± standard deviation (SD). Unpaired Student's *t*‐test was used to compare differences between two groups, while one‐way analysis of variance (one‐way ANOVA) and Tukey–Kramer post hoc test were employed for comparisons among more than two groups. Statistical significance was considered at *p* < 0.05.

## 3 Results

### 3.1 White Matter Injury in TLE Mice Indicated by LFB

**FIGURE 1:**
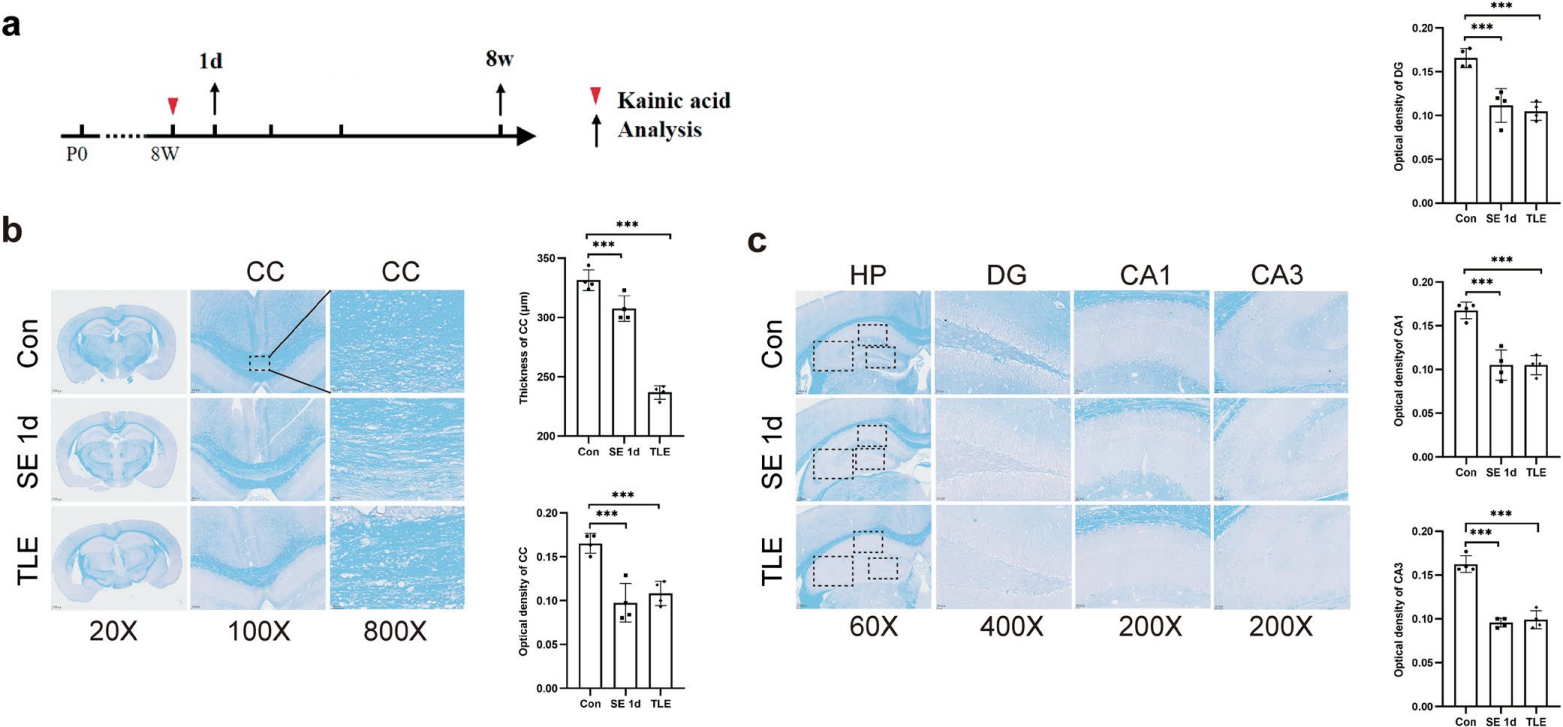
Changes in the myelin sheath in the hippocampus and the corpus callosum of SE mice. (a) Timeline of TLE mouse model establishment and sample analysis. (b) LFB staining reveals demyelination alterations in the corpus callosum (CC) of SE mice. Dashed box indicates the approximate location of the magnified images in CC. (c) LFB staining reveals demyelination alterations in the hippocampus of SE mice. Dashed box indicates the approximate location of the magnified images in DG, CA1, CA3 regions. Quantitative analysis (mean ± SEM, *n* = 4) showed significant differences by one‐way ANOVA (*p* < 0.05). Post hoc Tukey HSD tests indicated specific group differences (***p < 0.001).

As depicted in Figure 1, Mice underwent modeling at 8 weeks of age. Tissue samples were collected at 1 day post‐SE, and during the chronic TLE phase (8 weeks post‐modeling) (Figure 1a). LFB staining results revealed significant atrophy and thinning of the corpus callosum following SE, with these changes becoming more pronounced during the chronic phase. Additionally, vacuolation and disorganization were observed in myelin cells at 1 day post‐SE and in the TLE group, indicative of myelin injury (Figure 1b). In the hippocampus, myelin staining intensity in the DG, CA1, and CA3 regions appeared diminished, with cells exhibiting a loose and disorganized arrangement. Furthermore, OD values in the DG, CA1, and CA3 regions were significantly reduced after SE, demonstrating notable differences. These findings suggest that myelin damage occurs across all hippocampal regions following SE (Figure 1c).

### 3.2 Augmented Expression and Phosphorylation of Cx47 in TLE Mice and Human

**FIGURE 2:**
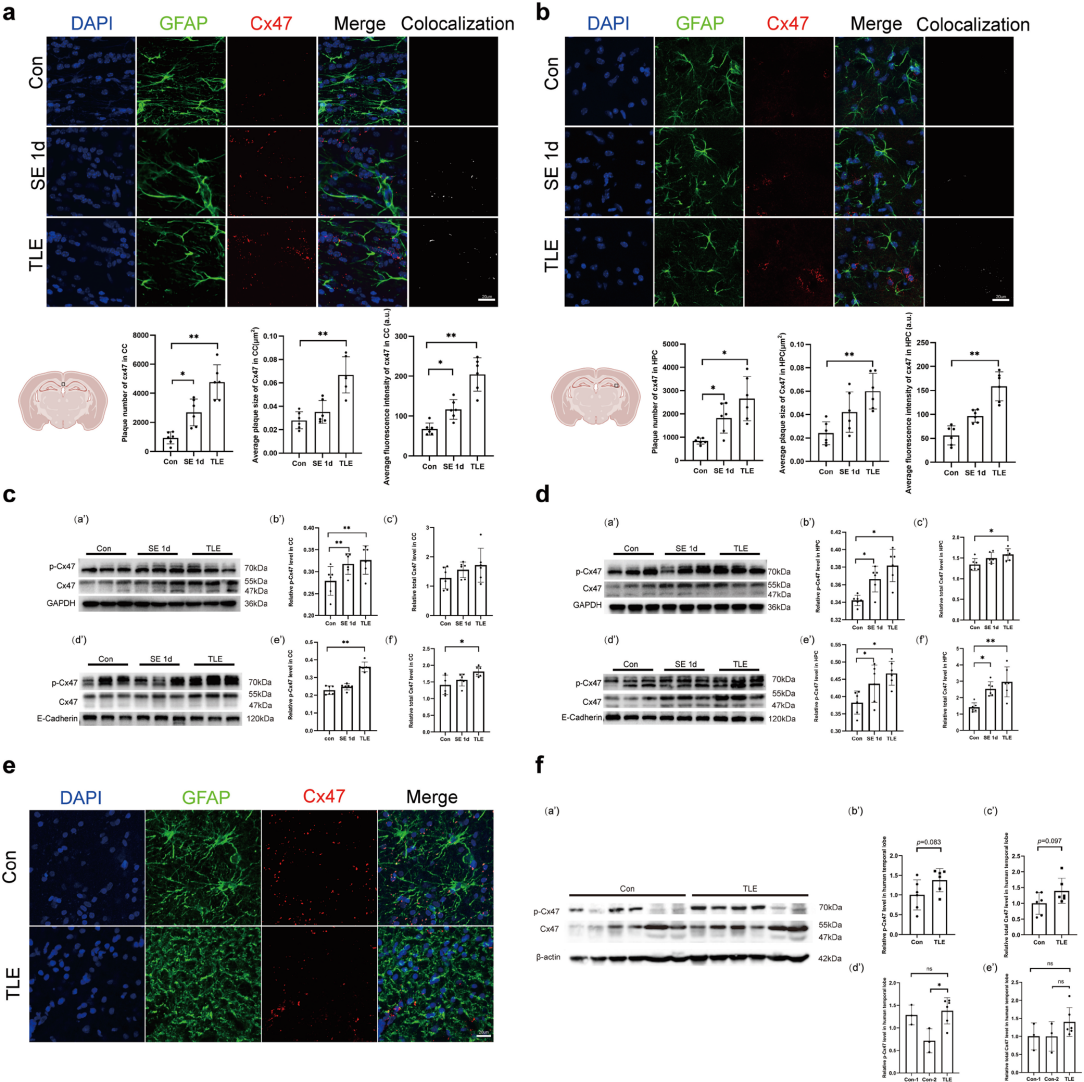
Changes in Cx47 expression in mouse brain tissue and patient brain tissue. (a) Changes in Cx47 expression in the CC of mouse brain tissue as detected by IF, where only colocalization signals are used for statistical analysis. Scale bar: 20 μm. (b) Changes in Cx47 expression in the hippocampus (HPC) of mouse brain tissue as detected by IF, where only colocalization signals are used for statistical analysis. Scale bar: 20 μm. (c) Variations in Cx47 expression in total and membrane protein fractions of the CC of mouse brain tissue: (a′–c′) total protein, (d′–f′) membrane protein. (d) Variations in Cx47 expression in total and membrane protein fractions of the HPC of mouse brain tissue: (a′–c′) total protein, (d′–f′) membrane protein. Quantitative analysis (mean ± SEM, *n* = 6) showed significant differences by one‐way ANOVA (*p* < 0.05). Post hoc Tukey HSD tests indicated specific group differences (*p < 0.05, **p < 0.01). (e) Typical IF images of Cx47 expression in the control group and TLE patient brain tissue. Scale bar: 20 μm. (f) Total protein changes in Cx47 protein expression in the control group and the TLE patient brain tissue, normalization was performed to Cx47 expression in the control group. (a') Representative WB of Cx47,p‐Cx47 and β ‐ actin. (b′, c′) Total and p‐Cx47 levels in controls and TLE (unpaired *t*‐test; mean ± SEM, *n* = 6). (d′, e′) Subgroup analysis (Con‐1, *n* = 3; Con‐2, *n* = 3; TLE, *n* = 6) by one‐way ANOVA with Tukey's post hoc test (overall *p* < 0.05; Con‐2 vs. TLE, *p < 0.05; all other comparisons; ns, not significant).

As illustrated in Figure 2, IF analysis of Cx47 and GFAP revealed a significant increase in Cx47 levels in the corpus callosum and hippocampal CA3 region in TLE group. A similar but less pronounced increase was also observed in the SE 1d group. This increase was primarily characterized by a rise in the number of Cx47 plaques, plaque area, and average fluorescence intensity (Figure 2a,b). Since phosphorylated Cx47 bands are poorly characterized in prior studies, the elimination and change of higher molecular weight bands (> 70 kDa) following alkaline phosphatase (ALP) treatment strongly supports their identification as phosphorylated isoforms of Cx47 (Figure S1). WB analysis indicated that the expression of total Cx47 and phosphorylated Cx47 (p‐Cx47) in the corpus callosum showed an upward trend during the acute phase of SE. However, the expression levels were more significant in the chronic phase, particularly for p‐Cx47 expression (Figure 2c). In the hippocampus, the expression of both total Cx47 and p‐Cx47 was increased during both the acute phases of SE and TLE. Specifically, the membrane expression of p‐Cx47 was markedly elevated in both SE 1d and TLE mice compared to the control group (Figure 2d).

In human brain tissue specimens, IF staining indicated that the colocalization level of Cx47 in patients with TLE has no difference compared to the control group. However, Cx47 plaques were observed to be significantly larger in TLE specimens relative to controls (Figure 2e). Furthermore, chronic dense fibrillary gliosis was identified in TLE specimens, aligning with previous research findings [38]. To control for connexin expression variability across pathologies [8], we analyzed resected temporal lobes from hemorrhagic patients as the Con‐2 group. WB showed elevated p‐Cx47 in TLE versus the control (Con‐2, *p* < 0.05). A comprehensive trend analysis across all control groups (including hemorrhagic diseases, cysts, and tumor‐related pathologies such as meningioma and glioblastoma) further indicated an upward regulatory pattern of this connexin in epileptogenic tissue (Figure 2f).

### 3.3 ScRNA‐Seq Reveals the Potential Association of *Gjc2* (Cx47) and *Calm1* (CaM), *Camk2b* (CaMKIIβ)

**FIGURE 3:**
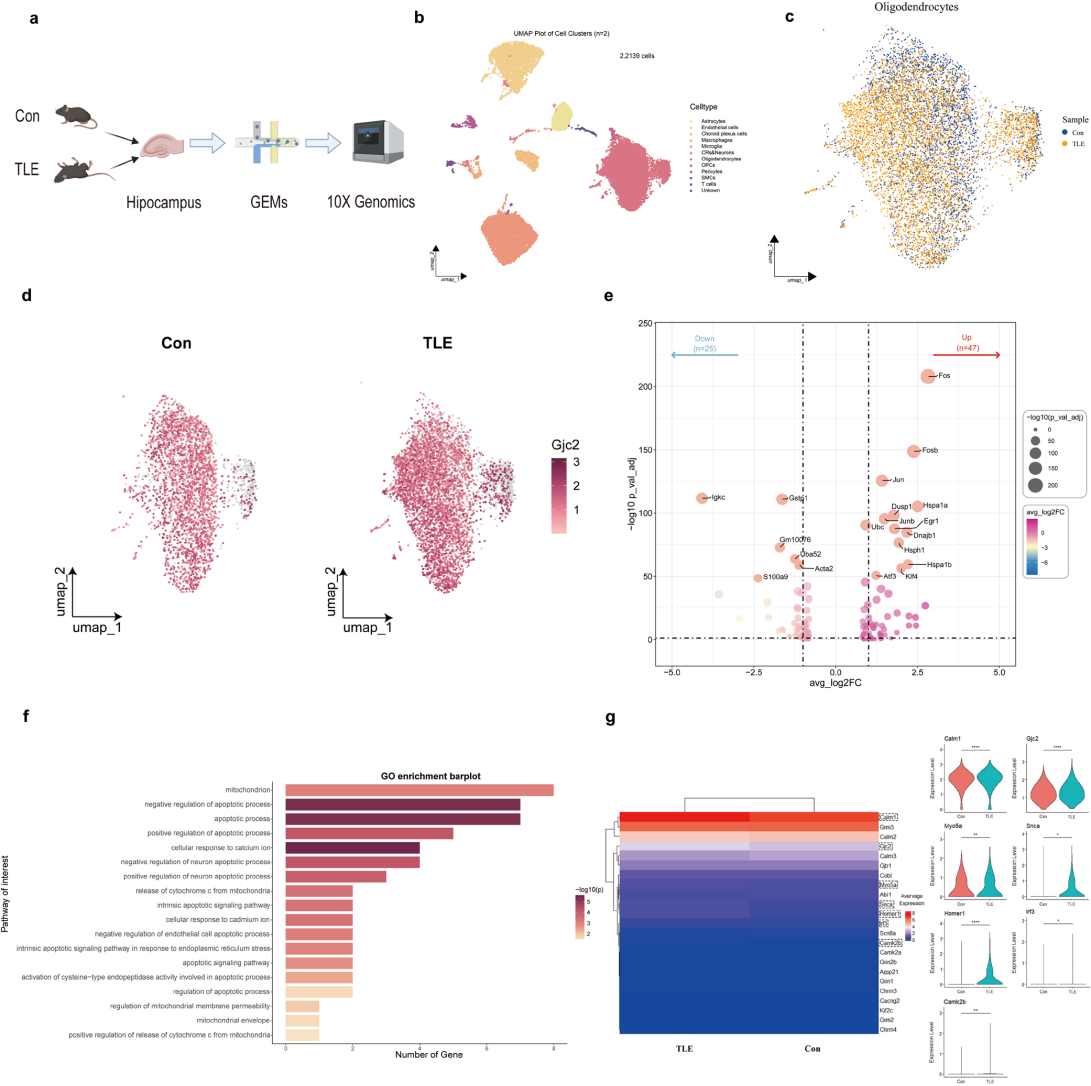
ScRNA‐Seq reveals the potential association of *Gjc2* (Cx47) and *Calm1* (CaM), *Camk2b* (CaMKIIβ). (a) Schematic of scRNA‐seq using the hippocampus of mice from TLE and control (Con) (*n* = 3). (b) Unsupervised clustering of cell populations and annotation of cell types. A total of 12 cell types were identified (CRs, Cajal–Retzius cells; OPCs, oligodendrocyte precursor cells; SMCs, smooth muscle cells). (c) The distribution of OL in different groups. (d) The relative expression and distribution of *Gjc2* in OL. (e) The Volcano plot depicting differentially expressed genes (DEGs) associated with *Gjc2* + OL between two groups (TLE vs. Con, |avg_log_2_FC| > 1, *p*_val_adj < 0.05). Dot size corresponds to the −log_10_(*p*_val_adj), color represents the average log_2_Foldchange. (f) The bar plot illustrating gene ontology (GO) enrichment of the interested pathway (*p* < 0.05). Bar length corresponds to the −log_10_(*p*), color represents the enrichment number of genes in the pathway. See Table S3 for more enrichment analyses. (g) The heatmap plot and violin plot of the GeneMANIA gene list. Color represents the average expression of genes (difference by Wilcoxon signed‐rank test, *p < 0.05, **p < 0.01, ***p < 0.001, ****p < 0.0001).

A total of 22,139 hippocampal cells were sequenced, comprising 11,707 cells from the TLE group and 10,432 cells from the control group. Quality control was performed based on the number of genes, molecules per single cell, mitochondrial gene ratio, and ribosome gene ratio in both groups. After filtering out low‐quality cells, clustering analysis identified 12 major cell types using key representative marker genes (Figure 3a,b and Figure S2).

Interestingly, the proportion of OL cells in the TLE group was 45.2% (5292/11,707), significantly higher than the 30.9% (3219/10,432) observed in the control group. The distribution of OL cells also exhibited distinct patterns in the UMAP visualization (Figure 3c), suggesting a functional shift in OL during the disease. Analysis of *Gjc2* mRNA expression and distribution in OL revealed a significant increase in the TLE group, consistent with our WB results. Additionally, the distribution of *Gjc2* was heterogeneous (Figure 3d), indicating that the expression pattern of *Gjc2* may influence OL pathology following TLE.

Further comparison of *Gjc2*‐positive OL between the two groups revealed that differentially expressed genes (DEGs) in the TLE group were enriched in pathways related to apoptosis, cell responsiveness to Ca^2+^, and mitochondrial function (Figure 3e,f, Tables S2 and S3). This suggests that *Gjc2*‐positive OL in the TLE group may upregulate apoptotic pathways, which we have previously demonstrated, and are linked to Ca^2+^ responsiveness. Previous studies have also implicated the CaM/CaMKII pathway in OL injury and modulation of MBP protein expression [39, 40]. Although no key genes of CaM/CaMKII were identified among the DEGs, we hypothesize that their upstream position may account for the lack of drastic changes. To explore this further, we expanded our gene panel by querying GeneMANIA using *Gjc2*, *Calm1*, *Camk2a*, and *Camk2b*, resulting in a list of genes of interest (Table S4). Heatmap and violin plots revealed that 7 out of 24 genes exhibited higher expression in the TLE group, including increased mRNA levels of *Calm1* (CaM) and *Camk2b* (CaMKIIβ) (Figure 3g).

### 3.4 ScRNA‐Seq Reveals the Dynamic Change in OL Lineages With *Gjc2*‐CaMKII‐Linked Calcium Signaling

**FIGURE 4:**
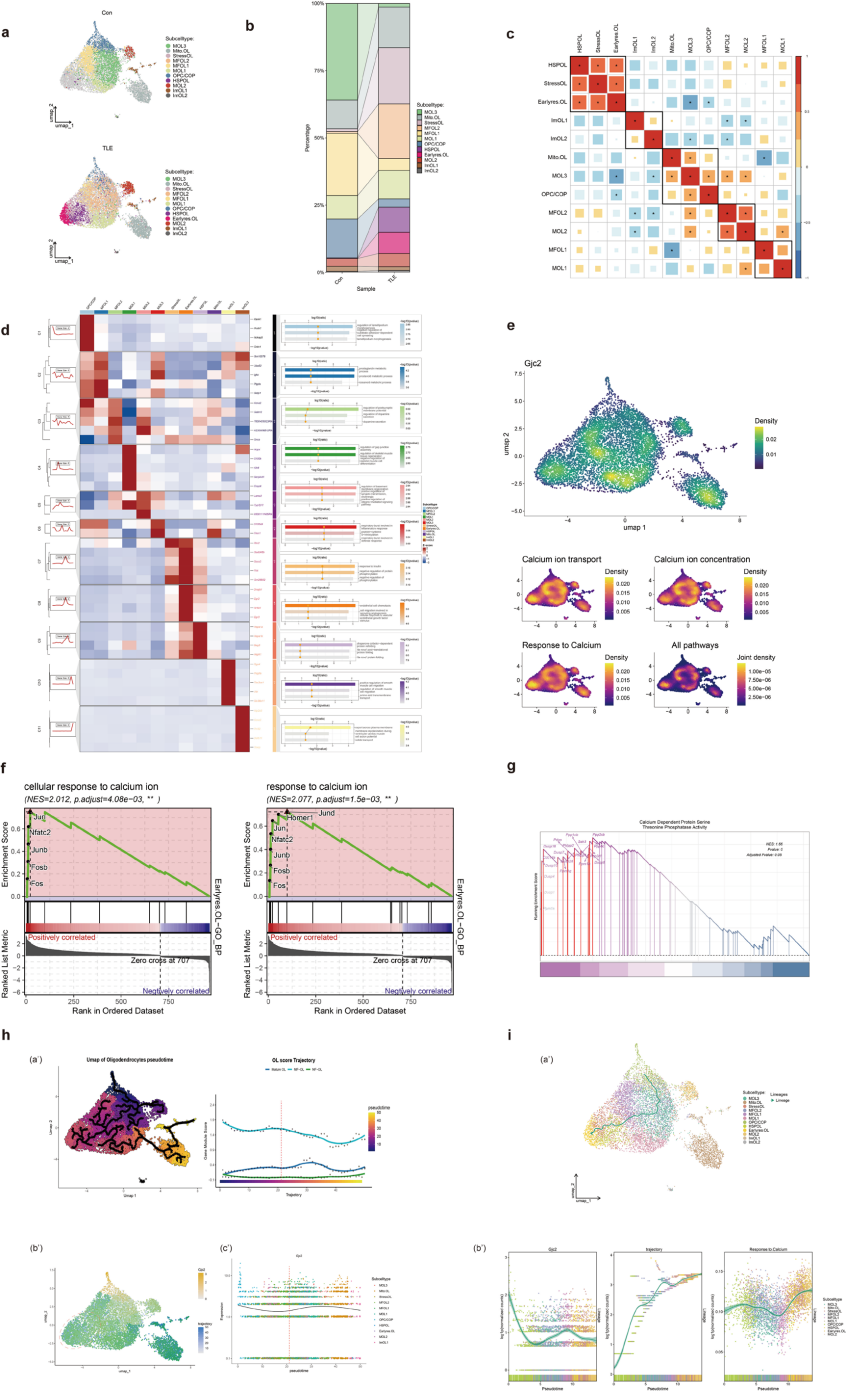
OL lineage subcelltypes response to TLE revealed by GSEA and pseudotime analysis. (a) Unsupervised clustering of cell populations and annotation of OL lineage's cell types. A total of 12 subcelltypes were identified (Earlyres.OL, early‐response oligodendrocyte; HSPOL, heat shock protein‐related oligodendrocyte; ImOL, immunity oligodendrocyte; MFOL, mature forming oligodendrocyte; Mito.OL, mitochondria‐related oligodendrocyte; MOL, mature oligodendrocyte; OPC/COP, oligodendrocyte precursor cell and committed oligodendrocyte precursor cell; StressOL, stress‐related oligodendrocyte). (b) The change of percentage between different sample groups. (c) The correlation analysis between subcelltypes (color represents the Pearson correlation coefficient. Statistically significant correlations are indicated with *p < 0.05). (d) The top 5 markers of each subcelltype and their relative top 3 GO_BP enrichment results. (e) The density UMAP of *Gjc2* expression and the AUCell score for calcium‐response pathways. (f) GSEA enrichment plots of Earlyres.OL, normalized enrichment score (NES) to estimate GSEA enrichment (**p.adjust < 0.01). (g) GSEA enrichment plots of StressOL, normalized enrichment score (NES) to estimate GSEA enrichment. (h, a′) The UMAP ofOL pseudotime and the dynamics of mature OL (mature oligodendrocyte), MF‐OL (mature forming oligodendrocyte), NF‐OL (newly forming oligodendrocyte) scores along the trajectory. The red dashed line indicates the transition point where the MF‐OL and mature OL signature scores intersect/reverse. (b′) The UMAP of OL pseudotime overlayed with *Gjc2* expression. The color of green represents the overlap of their density, red dashed circle represents the medium *Gjc2* expression and the middle trajectory of OL. (c′) The scatter plot of *Gjc2* expression along trajectory with a fitted regression line. The red dashed line indicates the transition point where *Gjc2* upregulation begins. (i, a′) The lineage of trajectory similar to Monocle 3 using Slingshot method. (b′) The scatter plot of *Gjc2* expression, Monocle 3 trajectory, AUCell score of Response to calcium pathway along trajectory with a fitted regression line.

We extracted oligodendrocyte precursor cells (OPCs) and OL from the scRNA sequencing dataset and performed dimensionality reduction and reclustering, and identified 12 distinct OL lineage subclusters [41], including OPC/COP, MFOL1–2, MOL1–3, mitochondria‐related OL (Mito.OL), stress‐related OL (StressOL), heat shock protein‐related OL (HSPOL), early‐response OL (Earlyres.OL), and ImOL1–2 (Figure 4a,d). Cell marker analysis showed that the three OL groups had distinct characteristics. StressOL carried markers associated with mitochondrial and ER stress regulation (Figures S3 and S4). HSPOL were enriched in heat shock protein family genes, while Earlyres.OL expressed immediate early genes, including *Fos*, *Jun*, and *Egr1* [42]. MOL1 resembled the human Oligo5 subtype, while MOL3 resembled human Oligo2 cluster [43]. Mito.OL expressed markers related to young‐related OL cluster [44] (Figure S5). Functional annotation revealed distinct phenotypes: Mito.OL expressed mitochondrial‐associated genes but showed reduced mitochondrial function (Figures S6–S9). Comparison before and after epilepsy induction showed significant expansion of StressOL, MFOL2, MOL1, HSPOL, Earlyres.OL, and MOL2, with StressOL and MFOL2 being most prominent. Notably, HSPOL, Earlyres.OL, and StressOL were nearly absent under baseline conditions but enriched after epilepsy onset. UMAP and correlation analyses confirmed their transcriptional proximity, suggesting they are epilepsy‐specific OL subtypes (Figure 4b,c).

We next examined *Gjc2* expression across OL subclusters. *Gjc2* was highest in MOL1, followed by Mito.OL and MFOL1, and moderately expressed in HSPOL, Earlyres.OL, and StressOL (Figure 4e). Calcium pathway scoring, based on epilepsy‐induced gene sets, paralleled *Gjc2* distribution, with the strongest enrichment in HSPOL, Earlyres.OL, and StressOL. GSEA revealed distinct calcium‐related pathway activities across these clusters: “response to calcium” and “cellular response to calcium” were specifically upregulated in Earlyres.OL. In contrast, these pathways were most downregulated in MOL1. Additionally, Stress‐OL showed significant upregulation of the “calcium‐dependent protein serine‐threonine kinase activity” pathway (Figure 4f,g, Figures S10 and S11). These findings suggest that HSPOL, Earlyres.OL, and StressOL mediate early calcium signaling responses, while MOL1 contributes to later phases, possibly through CaMKII‐related pathways.

Pseudotime analysis using Monocle3 identified OPC/COP as the lineage root. Oligodendrocyte lineage scores showed that NFOL and MFOL scores declined, while Mature‐OL scores increased along pseudotime. After pseudotime point 20, a marked inflection coincided with the emergence of HSPOL, Earlyres.OL, and StressOL. *Gjc2* expression subsequently entered a renewed upregulation phase, demonstrating a biphasic pattern with a second peak at approximately pseudotime 32–33 (Figure 4h, Figure S12).

Slingshot analysis confirmed similar lineage trajectories, showing OPC/COP progressing through MFOL2 and MOL3 toward HSPOL, Earlyres.OL, and StressOL. *Gjc2* peaked in MOL1 and MFOL1 but remained moderate in terminal subtypes, consistent with their differentiation states. Terminal clusters exhibited sustained calcium signaling enrichment, supporting their role in epilepsy‐associated OL remodeling (Figure 4i, Figure S13).

Additionally, functional enrichment revealed sequential transitions from neuronal projection and myelination to calcium and metal ion responses, linking *Gjc2* (Cx47) upregulation with OL re‐differentiation under epileptic conditions (Figure S14).

### 3.5 Multiple Lines of Evidence Demonstrate an Interaction Between Cx47 and CaMKII

**FIGURE 5:**
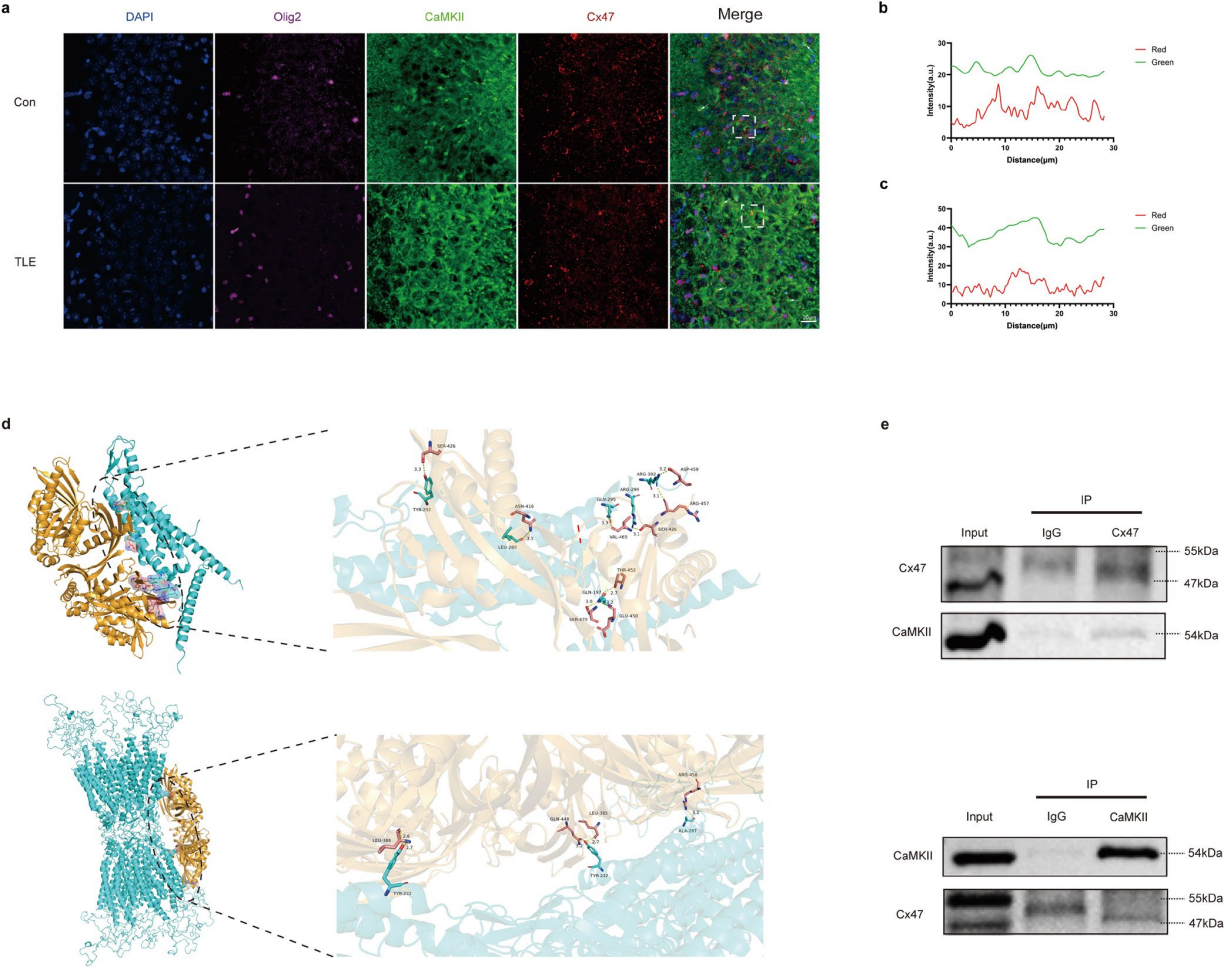
IF, docking, and Co‐IP reveal an interaction between Cx47 and CaMKII. (a) Typical IF images showing the co‐expression of Cx47, CaMKII, and Olig2 in the hippocampal CA3 area of control and TLE mice. Sections were co‐stained for the oligodendrocyte lineage marker Olig2 (magenta), CaMKII (green), Cx47 (red), and DAPI (blue, nuclei). The white boxes in the merge panels indicate regions shown for line‐scan analysis in b and c. Arrows highlight representative colocalizations of CaMKII and Cx47. Scale bar: 20 μm (all). (b, c) Line‐scan analysis quantifying the fluorescence intensity profiles of CaMKII (green line) and Cx47 (red line) in Con (b) and TLE (c) groups. (d) Predicted structural model of the Cx47‐CaMKII protein complex. The upper panels are the monomer Cx47 protein interacting with CaMKII. The lower panels are the analogous 12‐mer Cx47 protein interacting with CaMKII. The overall complex (left panel) and a close‐up view of the key interacting interface (right panel) are shown. Putative critical amino acid residues involved in the interaction are highlighted (yellow for CaMKII, green for Cx47). Hydrogen bonds (yellow dashed line), bond lengths are listed in the figures. (e) Co‐IP assays validating the physical interaction between Cx47 and CaMKII. Western blots show: (Upper) IP performed using an anti‐Cx47 antibody, followed by immunoblotting. (Lower) reciprocal IP performed using an anti‐CaMKII antibody followed by immunoblotting. Input lanes represent total protein lysate. Normal IgG was used as a negative control for the IP.

IF of Cx47/CaMKII/Olig2 expression was conducted in the hippocampus CA3 area of the control and TLE mice. As shown in Figure 5a, colocalization of Cx47 and CaMKII signals were observed within Olig2‐positive cells, and line‐scan analysis quantifying the fluorescence intensity profiles of CaMKII and Cx47 indicate that in the TLE group, the colocalization effect was more pronounced (Figure 5a–c). Molecular docking has identified potential compliant docking models between human Cx47 monomer or 12‐mer structure with human CaMK2A and the putative critical amino acid residues. Molecular docking demonstrated high‐affinity binding for both the Cx47 monomer (score: −400.20) and the 12‐mer (score: −464.96), with correspondingly high confidence scores of 0.9933 and 0.9982. Notably, residues such as tyrosine were identified, a finding that is significant as they represent potential sites for kinase phosphorylation (Figure 5d). Co‐IP assays validated the physical interaction between Cx47 and CaMKII (Figure 5e).

### 3.6 Augmentation of CaM/CaMKII in the Hippocampus and Corpus Callosum of TLE Mice and Human

**FIGURE 6:**
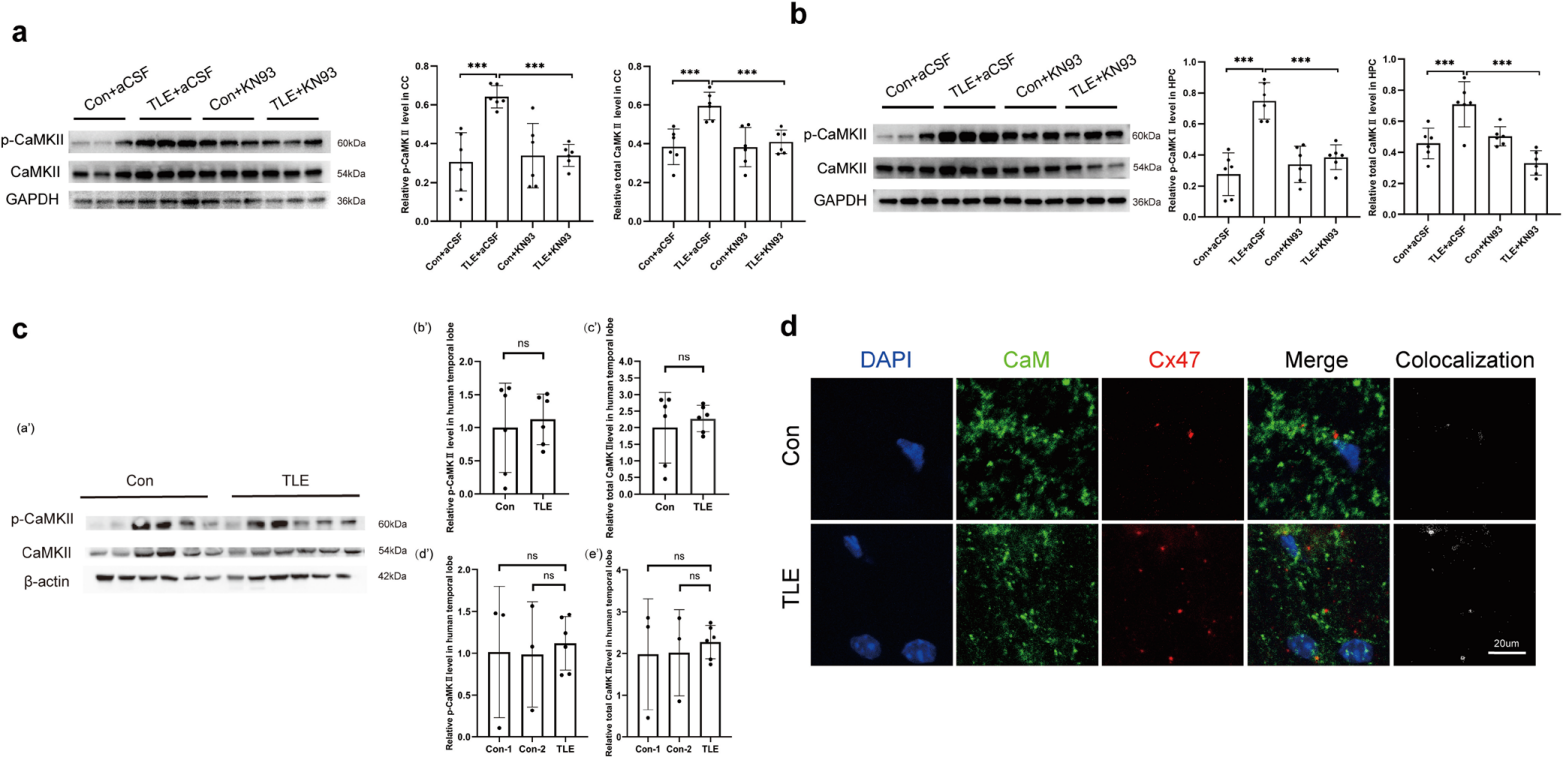
Changes in the expression and phosphorylation of CaMKII after KN93 administration. (a) WB analysis and quantification of CaMKII expression and phosphorylation in the CC total protein of mice. (b) WB analysis and quantification of CaMKII expression and phosphorylation in the HPC total protein of mice. Quantitative analysis (mean ± SEM, *n* = 6) showed significant differences by one‐way ANOVA (*p* < 0.05). Post hoc Tukey HSD tests indicated specific group differences (***p < 0.001). (c) WB analysis and quantification of CaMKII expression and phosphorylation in the human temporal lobe total protein, normalization was performed to CaMKII expression in the control group. (a') Representative WB of CaMKII, p ‐ CaMKII and β ‐ actin. (b′, c′) Total and p‐CaMKII levels in controls and TLE (unpaired *t*‐test; mean ± SEM, *n* = 6; ns, not significant). (d′, e′) Subgroup analysis (Con‐1, *n* = 3; Con‐2, *n* = 3; TLE, *n* = 6) by one‐way ANOVA with Tukey's post hoc test (ns, not significant). (d) Typical IF images of Cx47 expression around CaM in the control and TLE mice. Scale bar: 20 μm (all).

WB results demonstrated a significant increase in the expression of CaMKII and phosphorylated CaMKII (p‐CaMKII) in both the hippocampus and corpus callosum of TLE mice. This increase coincided with changes in p‐Cx47, suggesting that the phosphorylation of Cx47 may be associated with the elevated levels of CaMKII and p‐CaMKII (Figure 6a,b). Although TLE patients exhibited a nonsignificant trend toward elevated CaMKII and p‐CaMKII expression, this observation may be influenced by pathological alterations in control specimens (Figure 6c). Existing research has demonstrated that traumatic brain injury (TBI) can induce the activation of alpha‐CaMKII [45]. Notably, 33.3% (2/6) of control specimens originated from patients with TBI‐related pathologies, including subdural hematoma and traumatic subarachnoid hemorrhage.

Those supporting the potential link between these molecular changes and the pathophysiology of TLE. Additionally, the TLE model exhibited a significant increase in Cx47‐CaM colocalization density, suggesting that altered CaMKII signaling may be mechanistically linked to Cx47 expression dynamics (Figure 6d).

### 3.7 Reduced Phosphorylation of Cx47 by Inhibition of CaM/CaMKII Attenuates White Matter Injury and Seizure Severity in TLE Mice

**FIGURE 7:**
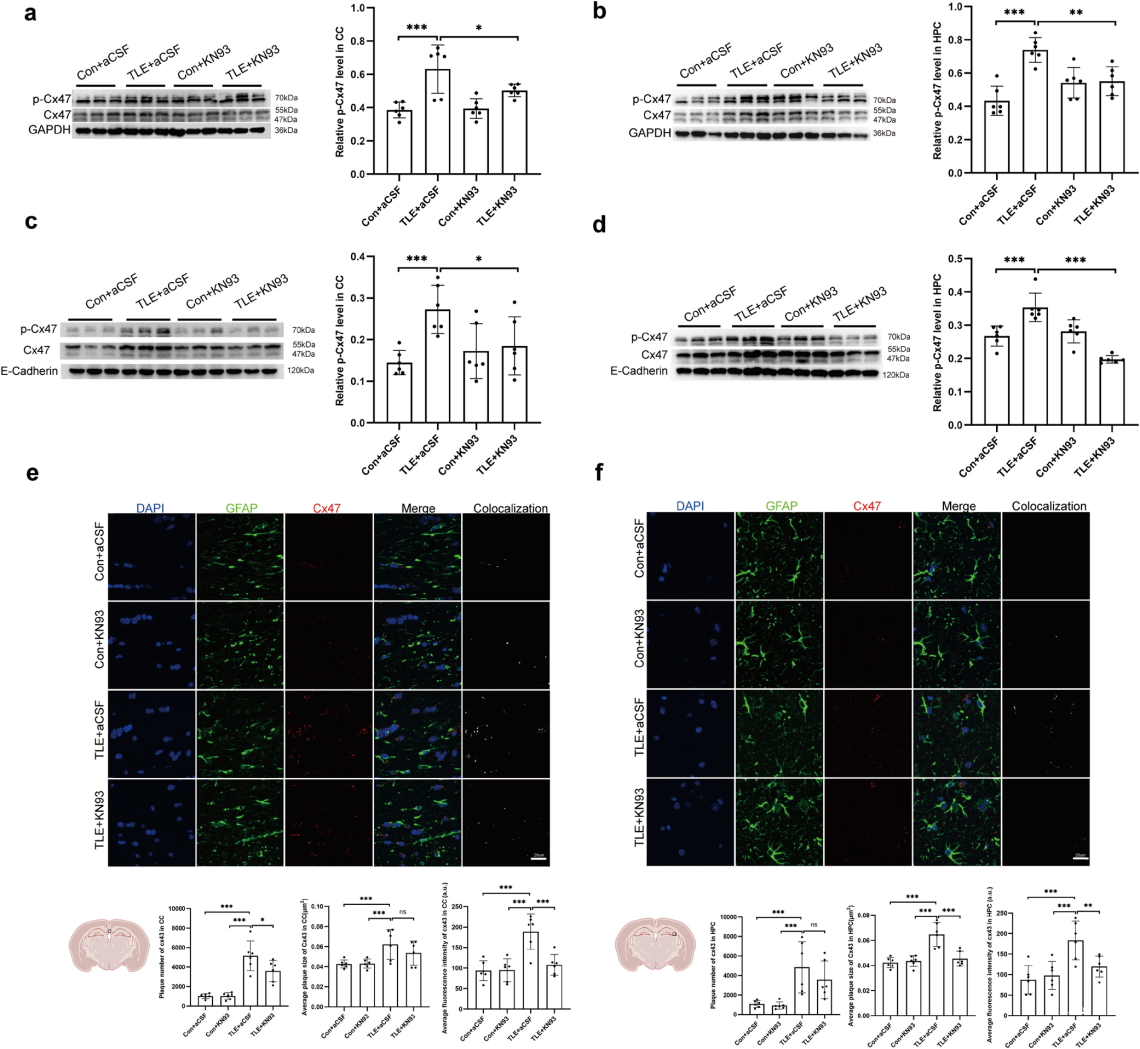
Changes in Cx47 expression in mouse brain tissue after KN93 administration. (a) WB analysis and quantification of Cx47 expression and phosphorylation in the CC total protein of mice. (b) WB analysis and quantification of Cx47 expression and phosphorylation in the HPC total protein of mice. (c) WB analysis and quantification of Cx47 expression and phosphorylation in the CC membrane protein of mice. (d) WB analysis and quantification of Cx47 expression and phosphorylation in the HPC membrane protein of mice. (e) Changes in Cx47 expression in the CC of mouse brain tissue detected by IF. (f) Changes in Cx47 expression in the HPC of mouse brain tissue detected by IF. Quantitative analysis (mean ± SEM, *n* = 6) showed significant differences by one‐way ANOVA (*p* < 0.05). Post hoc Tukey HSD tests indicated specific group differences (*p < 0.05, **p < 0.01, ***p < 0.001; ns, not significant).

Administration of KN93 partially inhibited the increase in CaMKII and p‐CaMKII levels (Figure 6a,b). WB and IF analyses revealed elevated expression of p‐Cx47 in the hippocampus and corpus callosum of TLE mice, which was attenuated following KN93 treatment (Figure 7).

**FIGURE 8:**
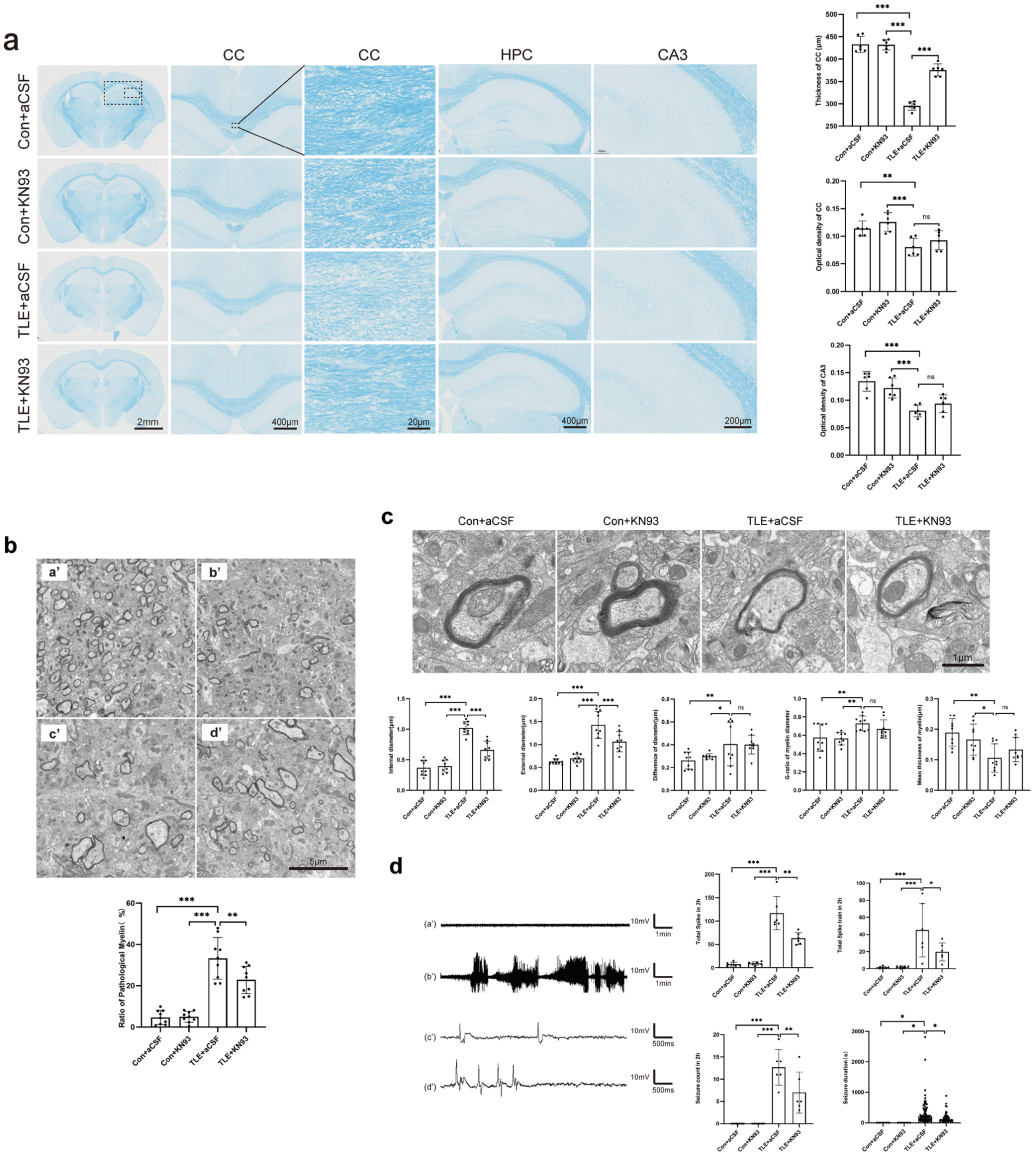
Changes in demyelination, myelin ultrastructural morphology, and seizure load after KN93 administration in mice. (a) LFB staining reveals demyelination alterations (*n* = 6). The dashed boxes indicate the regions shown at higher magnification below, corresponding to the entire HPC (large box), CA3 region (medium box), and CC (small box). (b) Ultrastructural myelin morphology and quantitative analysis. Representative TEM images from each group: (a′) Con + aCSF, (b′) KN93 + aCSF, (c′) TLE + aCSF, (d′) TLE + KN93. The bar graph shows the quantitative analysis of pathological myelin ratio (mean ± SEM, *n* = 9). (c) Representative ultrastructure of myelin sheaths and quantitative analysis. (d) Typical EEG waveforms and the seizure load statistical data of epileptic mice (*n* = 6). (a′) Baseline, (b′) epileptic seizure, (c′) spike, (d′) spike train. Quantitative analysis (mean ± SEM) showed significant differences by one‐way ANOVA (*p* < 0.05). Post hoc Tukey HSD tests indicated specific group differences (*p < 0.05, **p < 0.01, ***p < 0.001; ns, not significant).

LFB staining demonstrated that TLE mice exhibited thinning of the corpus callosum, which was partially reversed by KN93 treatment, although it remained thinner compared to controls. Myelin OD values in the corpus callosum and hippocampal CA3 regions were significantly reduced in TLE mice compared to controls, while no significant difference observed between the TLE + aCSF and TLE + KN93 groups (Figure 8a).

Transmission electron microscopy (TEM) analysis revealed more pronounced pathological changes in myelin, such as loosening, lamellar disruption, and vesicle formation, in the TLE + aCSF and TLE + KN93 groups compared to controls. KN93 intervention significantly reduced the rate of these pathological injuries relative to the TLE + aCSF group (Figure 8b). While KN93 decreased the inner and outer diameters of the myelin sheath in TLE mice, no significant differences in the G‐ratio were observed between the groups (Figure 8c).

EEG analysis indicated that TLE mice exhibited significantly more spikes, spike trains, and seizures, with prolonged seizure duration, compared to controls. KN93 treatment markedly reduced the number of spikes, seizures, and their duration (Figure 8d).

## 4 Discussion

This study revealed that upregulation and phosphorylation of Cx47 are associated with demyelination and OL apoptosis in TLE. Inhibiting the phosphorylation of Cx47 by reducing the expression and phosphorylation of CaMKII alleviated white matter injury, thereby ameliorating the seizure burden in TLE. These findings shed light on the mechanisms of epileptogenesis related to Cx47 dysregulation and demyelination.

We have previously reported that white matter injury, characterized by OL apoptosis in TLE, increases susceptibility to seizures [46]. Consistently, accumulating clinical data highlight pathological demyelination in various forms of epilepsy [47, 48]. Pelizaeus‐Merzbacher‐like disease, caused by biallelic pathogenic variants in the *GJC2* gene (encoding Cx47), is associated with hypomyelinating leukodystrophies observed on brain MRI, neurodevelopmental delay, and epilepsy in some cases [49, 50]. Thus, dysfunctional Cx47 in OL is presumed to contribute to demyelination and seizure progression in TLE.

Consistent with the upregulation of Cx47 expression observed in the local myelin defect regions of transgenic mice with Alzheimer's disease [51], both the total and membrane‐bound Cx47 protein levels were found to be elevated in a mouse model of TLE. Furthermore, Cx47 was identified as being phosphorylated and significantly increased in the hippocampi of TLE mice, even during the acute phase. This observation was corroborated by the detection of elevated phosphorylated Cx47 in human temporal lobe resection tissues. Zhao et al. [16] demonstrated that the calcium signaling pathway was dysregulated across all phases in Cx47‐deficient experimental autoimmune encephalomyelitis (EAE) mice, indicating a critical role for Cx47 in the regulation of calcium signaling. Similarly, Murugan et al. [52] reported that following hypoxic–ischemic brain injury, calcium signals were transmitted from AST to OL via GJ, with the resulting elevation in calcium ions leading to mitochondrial dysfunction and subsequent OL death. Given that astrocytic Ca^2+^ oscillations have been shown to lower the threshold for ictal activity during the early stages of epileptogenesis [8], it is plausible that demyelination in TLE may be attributed to an overload of calcium signaling molecules. This overload is likely mediated by alterations in coupling facilitated by Cx47‐containing gap junctions, particularly those between astrocytes and oligodendrocytes.

To investigate the alterations in the expression and phosphorylation of Cx47, we conducted scRNA‐seq on hippocampal tissue from a mouse model of TLE. The analysis revealed a significant upregulation of *Gjc2* mRNA, which encodes Cx47, in TLE mice. Concurrently, an increase in the mRNA levels of CaM and CaMKII was observed in *Gjc2* mRNA‐positive cells. This finding suggests that CaM and CaMKII may play a regulatory role in the phosphorylation of Cx47 and the apoptosis of OL in TLE. Consistent with this observation, both CaM and CaMKII expression levels were significantly elevated in TLE mice, with a similar trend identified in human tissue specimens. Despite the lack of statistical significance observed in the expression levels of both CaMKII and Cx47 in human tissue specimens, regional heterogeneity within the tissue samples may serve as a contributing factor. Notably, the control group comprised patients with TBI‐related pathologies (33.3%, 2/6), tumors (33.3%, 2/6), and other conditions (33.3%, 2/6). This compositional heterogeneity in the control cohort may have confounded baseline assessments of CaMKII/Cx47 expression patterns; therefore potentially diminishing intergroup differentials. These factors may collectively account for the observed variability in the expression patterns of CaMKII and Cx47.

Although Cxs lack sensitive intracellular Ca^2+^ binding sites for CaM, CaM has been proposed to act as an intermediate in the Ca^2+^ gating effect. It has been demonstrated that CaM can bind to the C‐terminal domain (CT1) of several connexins, including Cx35, Cx36, Cx34.7, and Cx46 in mice [53, 54, 55, 56, 57]. Furthermore, the formation of the Ca^2+^/CaM complex can activate downstream CaMKII, thereby influencing the functions of Cx36 in neurons and Cx43 in AST [58, 59, 60]. Notably, CaMKII undergoes autophosphorylation at T286 following activation by the Ca^2+^/CaM complex and subsequently binds to the CT1 binding site of Cx36, altering its phosphorylation state in Neuro2a cells. In this study, both the expression of CaMKII and its phosphorylation at T286 were significantly increased, paralleling the observed phosphorylation of Cx47. In addition, the results of Cx47/CaMKII/Olig2 IF, molecular docking, and Co‐IP assays all suggest that there is an interaction between Cx47 and CaMKII. Importantly, KN93, a competitive inhibitor of CaMKII, was found to reduce the levels of phosphorylated Cx47, ameliorate white matter injury, and alleviate seizure burden. Collectively, these findings suggest that CaMKII likely regulates the phosphorylation process of Cx47 in TLE.

The mechanism by which CaMKII regulates the phosphorylation of Cx47 remains to be fully elucidated. However, insights can be drawn from studies on related connexins. For instance, Cx36 has been shown to undergo phosphorylation at specific sites (S110 and T111) directly as a result of CaMKII activation [61]. Furthermore, the removal of CaMKII binding sites on the cytoplasmic loops of Cx36 [62] or the use of the CaMKII inhibitor KN93 [63] has been demonstrated to alter the conductivity of Cx36/Cx36 GJs. Similarly, Richard et al. identified 15 phosphorylation sites of CaMKII at the C‐terminal domain of Cx43, suggesting a more extensive regulatory role of CaMKII in connexin phosphorylation.

In this study, the synchronous changes in phosphorylated CaMKII (T286) and phosphorylated Cx47 indicate that CaMKII may also target specific phosphorylation sites on Cx47, analogous to its effects on Cx36 and Cx43. This observation raises the possibility that CaMKII‐mediated phosphorylation of Cx47 could modulate its function, potentially influencing GJ conductivity and cellular signaling in the context of TLE. Future investigations aimed at identifying the precise phosphorylation sites of Cx47 and their functional implications will be crucial to fully understand the regulatory role of CaMKII in this process.

The gap junction formed by Cx47/Cx43 heterotypic channels between AST and OL facilitates the passage of small molecules with a molecular weight < 1000 Da, including ATP, IP3, and calcium ions. An increase in astrocytic Ca^2+^ signals precedes ictogenesis and is regarded as an initiator of epileptogenesis [64]. It has been reported that Cx43/Cx47 GJ formed between the two was weakened after siRNA interfered with Cx47, and Ca^2+^ from AST to OPCs decreased significantly. This suggests that Cx47 can affect Ca^2+^ signal transmission from AST to OL by regulating the coupling function of Cx43/Cx47 [65]. These findings highlight the critical role of Cx47 in modulating intercellular communication and its potential implications in the pathophysiology of epilepsy. Further studies are needed to explore the precise mechanisms by which Cx47 influences GJ conductivity and Ca^2+^ signaling, particularly in the context of TLE.

Based on the observed increase in phosphorylation of Cx47 in both experimental TLE mouse models and clinical patient samples, it is plausible that this posttranslational modification plays a critical role in modulating the function of Cx47/Cx43 GJs. Phosphorylation of connexins is a well‐established regulatory mechanism that influences various aspects of GJ dynamics, including assembly, channel gating, degradation, and turnover. For instance, phosphorylation of Cx43 at Ser365 has been shown to prevent PKC‐mediated downregulation and stabilize GJ assembly, thereby ensuring efficient intercellular communication [66, 67]. Similarly, phosphorylation at specific residues, such as Ser368 on Cx43 by PKC, modulates the open probability of GJ channels, regulating electrical and metabolic coupling [66]. In immune cells, kinases such as BTK and ITK phosphorylate Cx43 at residues Y247, Y265, and Y313, akin to Src, Pyk2, and Tyk2, leading to reduced GJ intercellular communication and altered Cx43 function, highlighting cell‐specific kinase regulation of GJs [68]. Additionally, phosphorylation can promote connexin degradation and turnover by targeting connexons for endocytosis and lysosomal degradation, dynamically adjusting GJ communication to meet cellular needs [69].

Our study has several limitations that warrant discussion. First, the proposed interaction between CaMKII and Cx47 currently relies on indirect correlative evidence. To establish mechanistic causality, future studies should employ conditional knockout models (e.g., Olig2‐CreERT2; *Gjc2*‐floxed mice) combined with CRISPR‐Cas9‐mediated Cx47 deletion in OL lineage cells. Second, although altered Cx47 expression patterns were observed, their functional impact on AST/OL coupling remains unknown. Future studies should employ whole‐cell patch clamping and dye uptake to evaluate Cx47‐mediated coupling [19], particularly to test the functional impact of Cx47 phosphorylation on glial connectivity. Third, given that CaMKII constitutes ~10% of postsynaptic density proteins in hippocampal excitatory synapses [70], our exclusive focus on oligodendrocytic Cx47 regulation may overlook potential neuron‐autonomous effects.

These findings collectively underscore the importance of connexin phosphorylation in maintaining GJ function. Therefore, we hypothesize that the increased phosphorylation of Cx47 in TLE may enhance the coupling function of Cx43/Cx47 GJs, potentially contributing to the dysregulation of intercellular communication observed in epilepsy. However, the precise phosphorylation sites on Cx47, the specific kinases involved, and the downstream effects on GJ conductivity and Ca^2+^ signaling remain to be fully elucidated. Future studies aimed at identifying these mechanisms will be essential to understanding the role of Cx47 phosphorylation in the pathophysiology of TLE and to explore potential therapeutic targets.

## 5 Conclusion

Our findings demonstrate that the upregulation of p‐Cx47, mediated by CaM/CaMK‐dependent phosphorylation, is a significant contributor to seizure progression in TLE. We propose that this phosphorylation may enhance Cx47/Cx43 gap junction coupling, which in turn exacerbates seizure activity. Consequently, targeting Cx47 phosphorylation emerges as a promising therapeutic strategy, with the potential to restore intercellular communication between astrocytes and oligodendrocytes and thereby promote remyelination in TLE.

## Funding

This work was supported by the National Natural Science Foundation of China (no. 82071476) and the Chongqing Medical University Program for Youth Innovation in Future Medicine (grant number W0031).

## Ethics Statement

This study was performed in line with the principles of the Declaration of Helsinki. The human specimen approval was granted by Army Medical University Ethics Committee (approval number: 2022‐361‐01) and the animal experiment was approved by the Ethics Committee of Children Hospital of Chongqing Medical University (CHCMU‐IACUC20250407007).

## Consent

Written informed consent was obtained from the families.

## Conflicts of Interest

The authors declare no conflicts of interest.

## Supporting information


**Tables S1–S4:** cns70672‐sup‐0001‐TablesS1‐S4.xlsx.

**Figures S1–S14:** cns70672‐sup‐0002‐FiguresS1‐S14.pdf.

## Data Availability

The scRNA sequencing datasets generated during this study are not immediately publicly available, but can be obtained from the corresponding author upon reasonable request with appropriate data sharing agreements.
