# Supplementary material for: Cx47 Phosphorylation Exacerbates White Matter Damage and Kainic Acid Induced Epilepsy
Source: CNS Neurosci Ther. 2025 Nov 26;31(11):e70672. doi: 10.1111/cns.70672 (PMC12657264; doi:10.1111/cns.70672)
Supplement: Supplementary file 2 — Figures S1–S14: cns70672‐sup‐0002‐FiguresS1‐S14.pdf. [file CNS-31-e70672-s002.pdf]

Supplmentary Figures

Title: Cx47 phosphorylation exacerbates white matter damage and kainic acid induced epilepsy

Authors: Yi Li1,2,3#, Haohan Lin1,2#, Jiayu Liu1,2, Jie Chen1,2, Kaifeng Shen4, Ningning Chen1,2, Songyang Xiang1,2, Duan Wang1,2, Nong Xiao 1,2*, Ting song Li1,2*
Email of corresponding author: li.tingsong@cqmu.edu.cn; xiaonongwl@163.com
Affiliation:1 Department of Rehabilitation Children's Hospital of Chongqing Medical University,
National Clinical Research Center for Child Health and Disorders,
Ministry of Education Key Laboratory of Child Development and Disorders, Chongqing 40014, China
2 Chongqing Key Laboratory of Child Neurodevelopment and Cognitive Disorders, Chongqing 40014, China

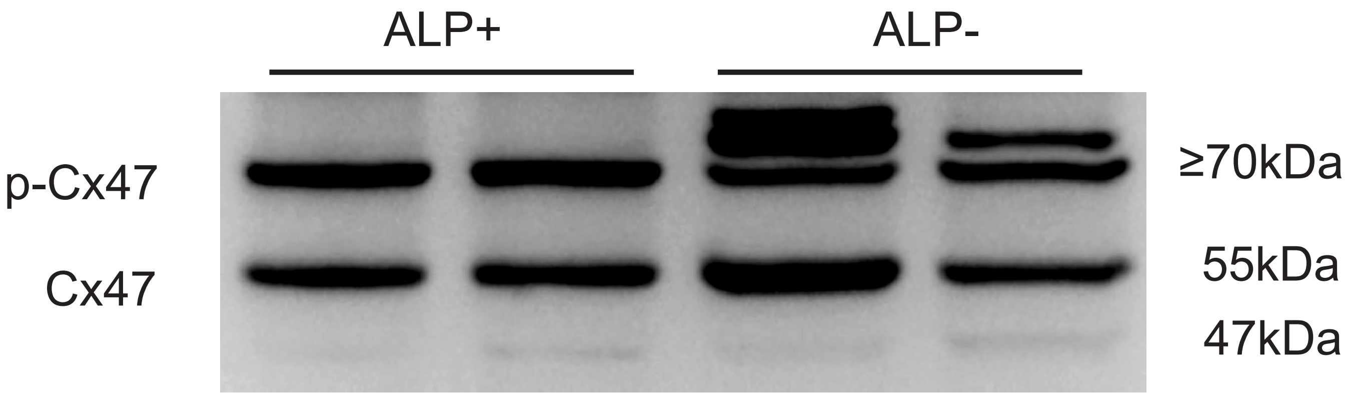

Supplmentary Figure 1.Following treatment with alkaline phosphatase (ALP), several bands detected by the Cx47 antibody above 70 kDa disappeared, while one band exhibited increased intensity.

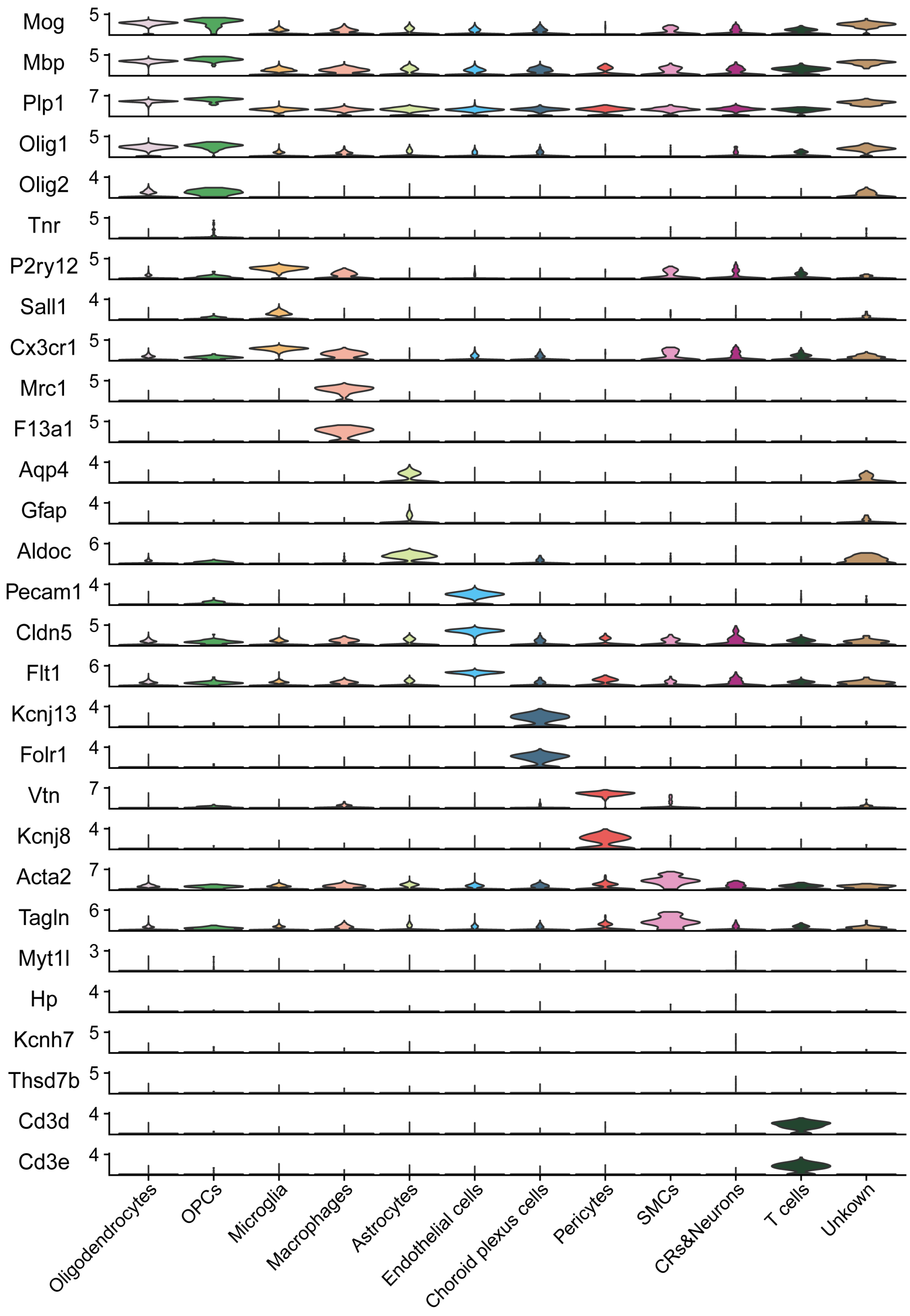

Supplmentary Figure 2. Vliolin plot of classified cell types according to the markers of each cell type.

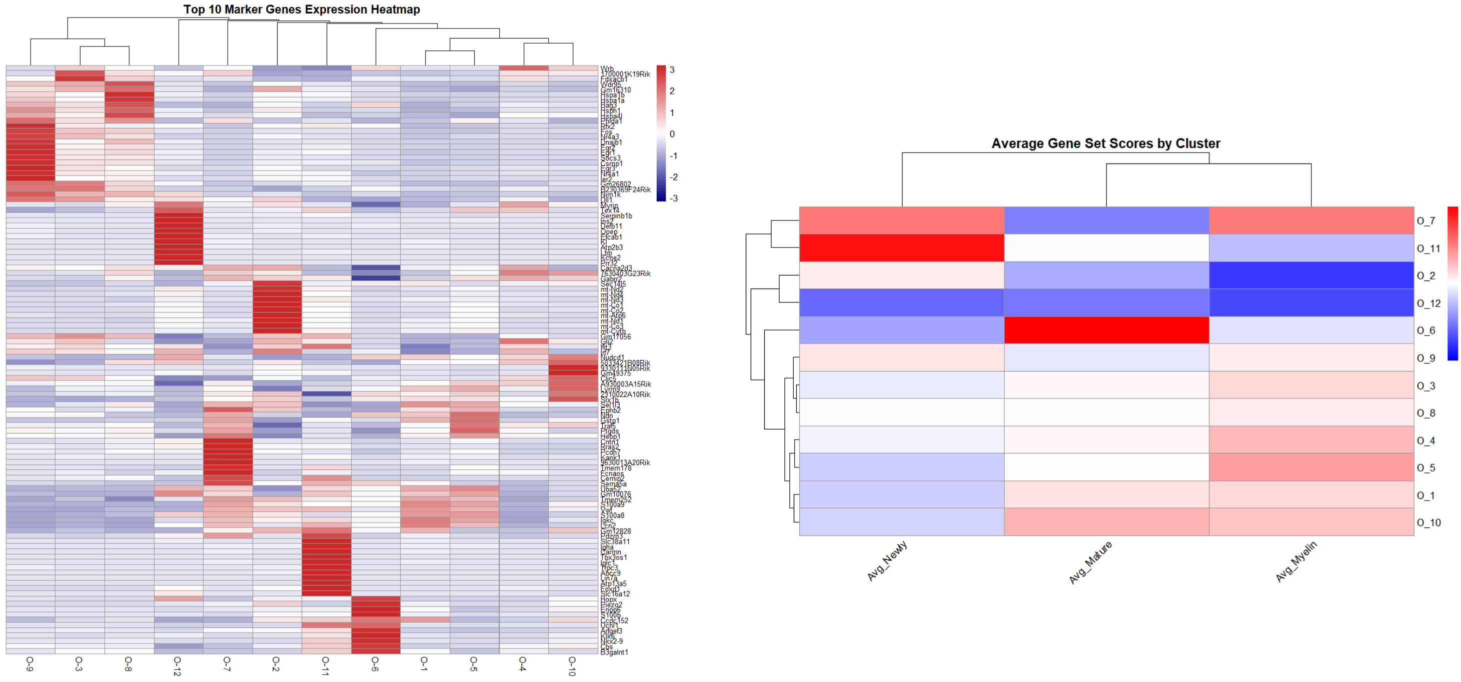

Supplementary Figure 3. Heatmap of top 10 marker genes and OL lineage signature scores across initial clusters.

The initial cluster designations (O_1 to O_12) correspond to the following subcelltype annotations:
O_1: MOL3, O_2: Mito.OL, O_3: StressOL, O_4: MFOL1, O_5: MFOL2, O_6: MOL1, O_7: OPC/COP, O_8: HSPOL, O_9: Earlyres.OL, O_10: MOL2, O_11: ImOL1, O_12: ImOL2.

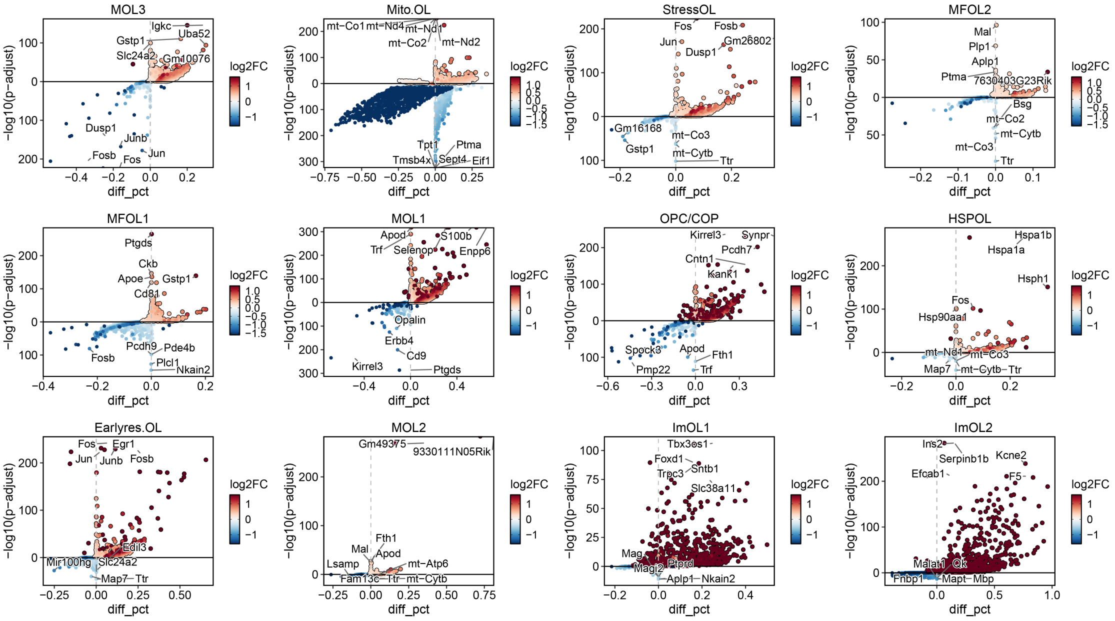

Supplementary Figure 4. Volcano plot depicting differentially expressed genes (DEGs) in different subcelltypes

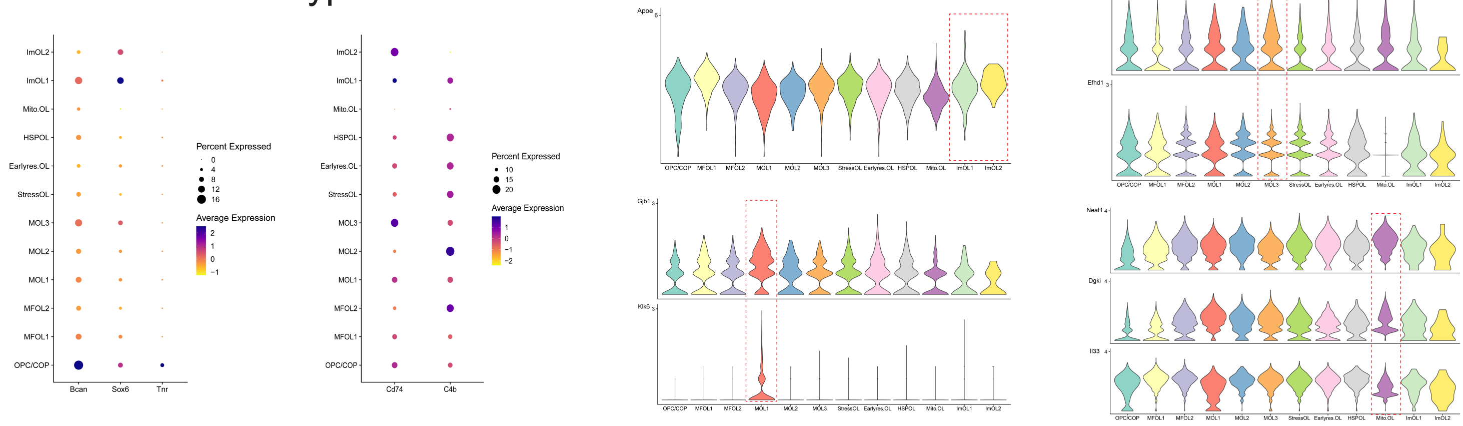

Supplementary Figure 5. Expression of oligodendrocyte lineage markers across subcelltype

Dot plots illustrate the expression of markers for OPC/COP (Bcan, Sox6, Tnr) and ImOL (Cd74, C4b).
Violin plots show the expression distribution of markers for ImOL, MOL1, MOL3, and Mito.OL, including Apoe(ImOL markers), Gjb1, Klk6 (Human Oligo5 markers), Cdh19, Efhd1 (Human Oligo2 markers), and Neat1, Dgki, Il33 (young-related oligodendrocyte markers in mice).

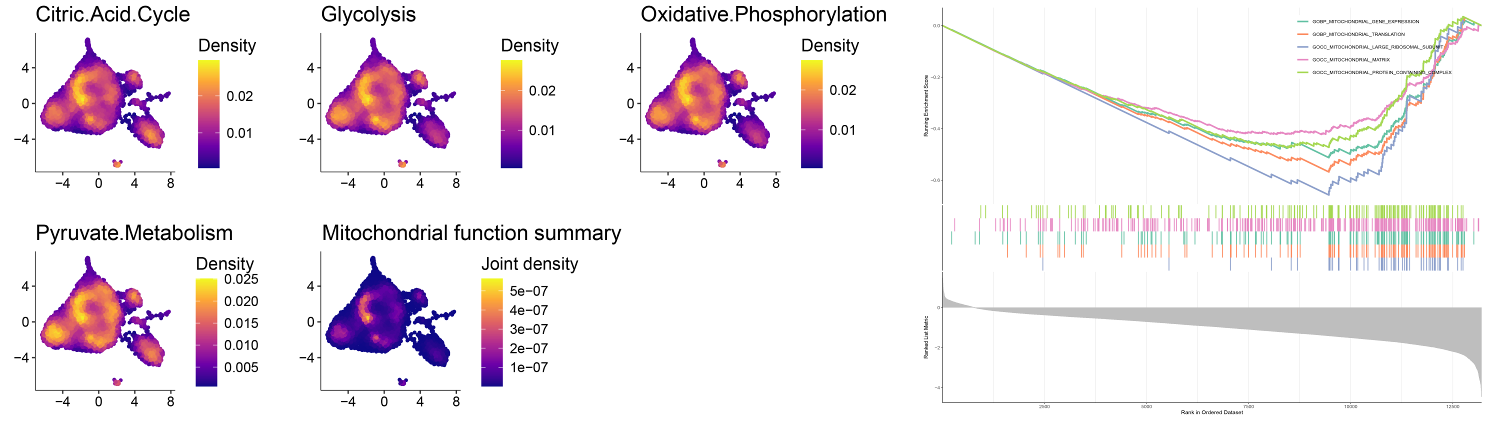

Supplementary Figure 6. Mitochondrial function and gene enrichment in OL

UMAP plots of OL are shown, with cells colored by AUCell scores reflecting mitochondrial function. Additionally, GSEA enrichment plot for Mito.OL is presented, utilizing mitochondria-related gene sets.
GSEA enrichment is estimated by the Normalized Enrichment Score (NES).

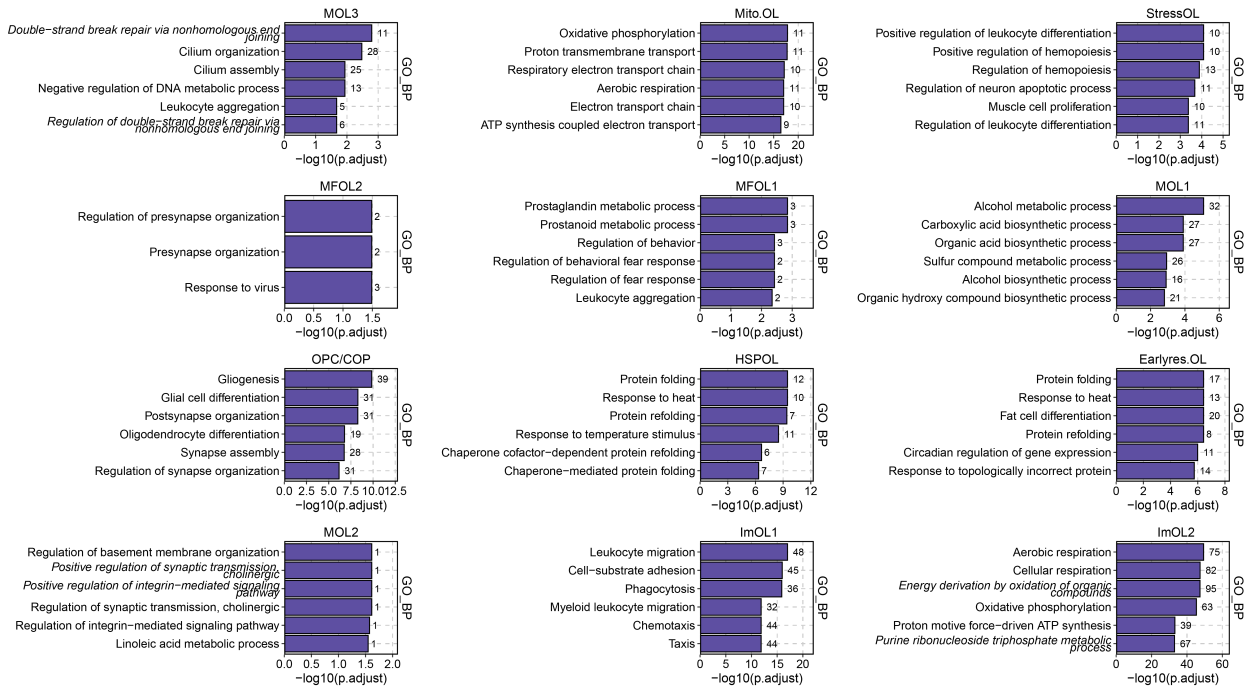

Supplementary Figure 7. Top 6 Gene Ontology Biological Process(GO_BP) enrichments for each subcelltype

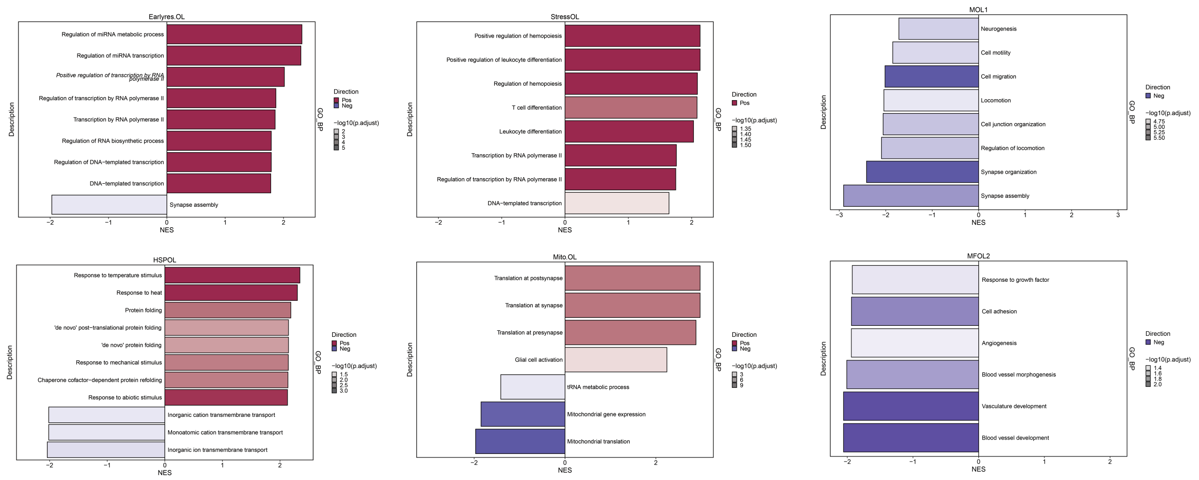

Supplementary Figure 8. Bar plot of top 15 Gene Set Enrichment Analysis(GSEA) enrichments in upregulated subcelltypes

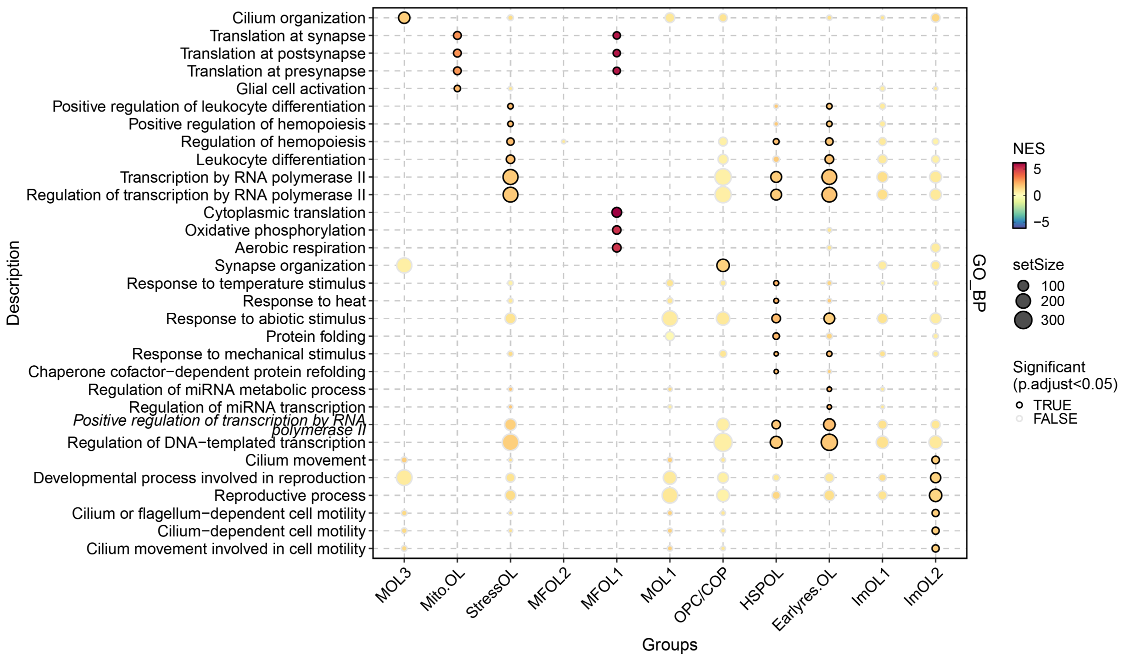

Supplementary Figure 9. Dot plot of GSEA enrichments across all subcelltypes

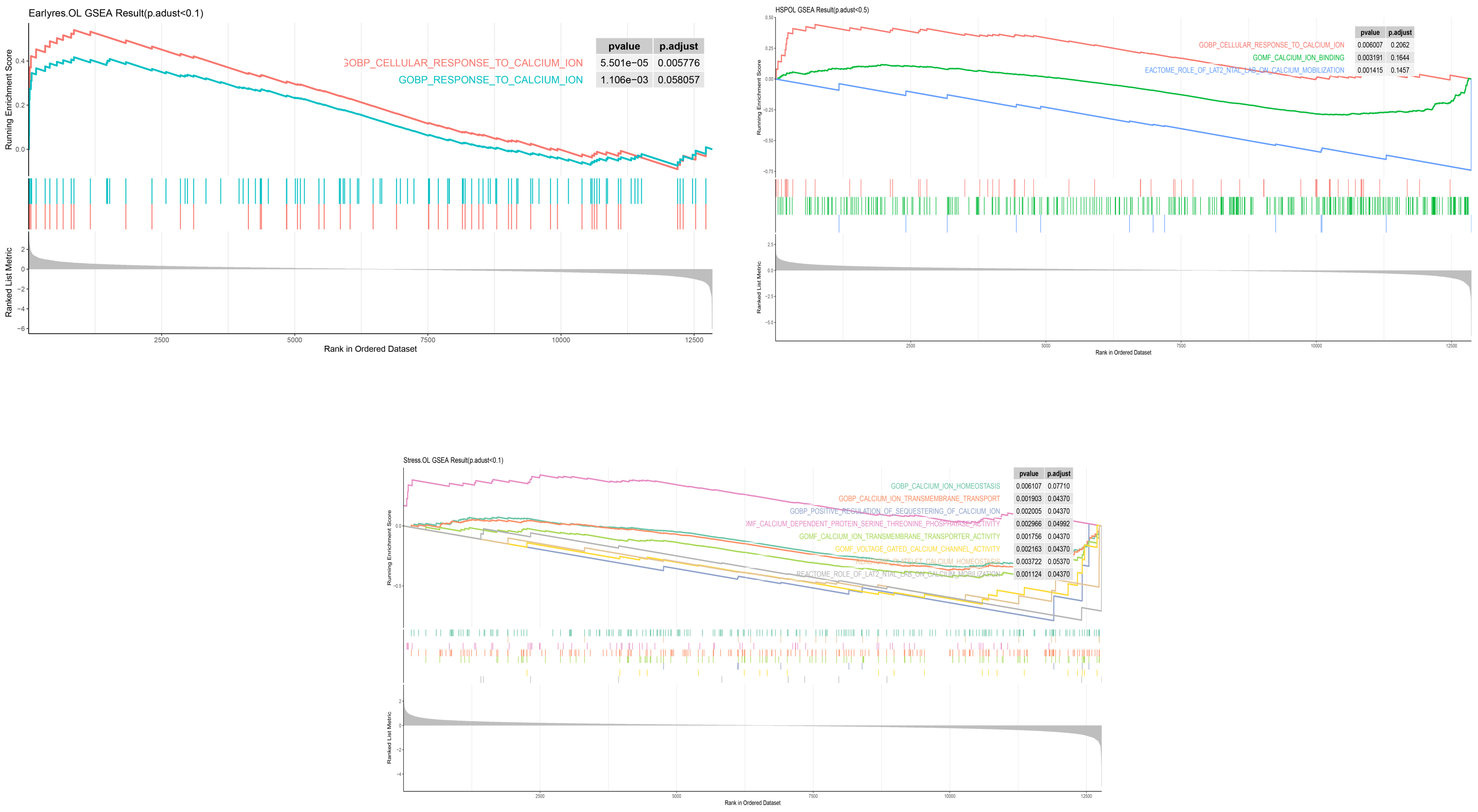

Supplementary Figure 10. GSEA plots for specific oligodendrocyte populations using calcium-related gene sets

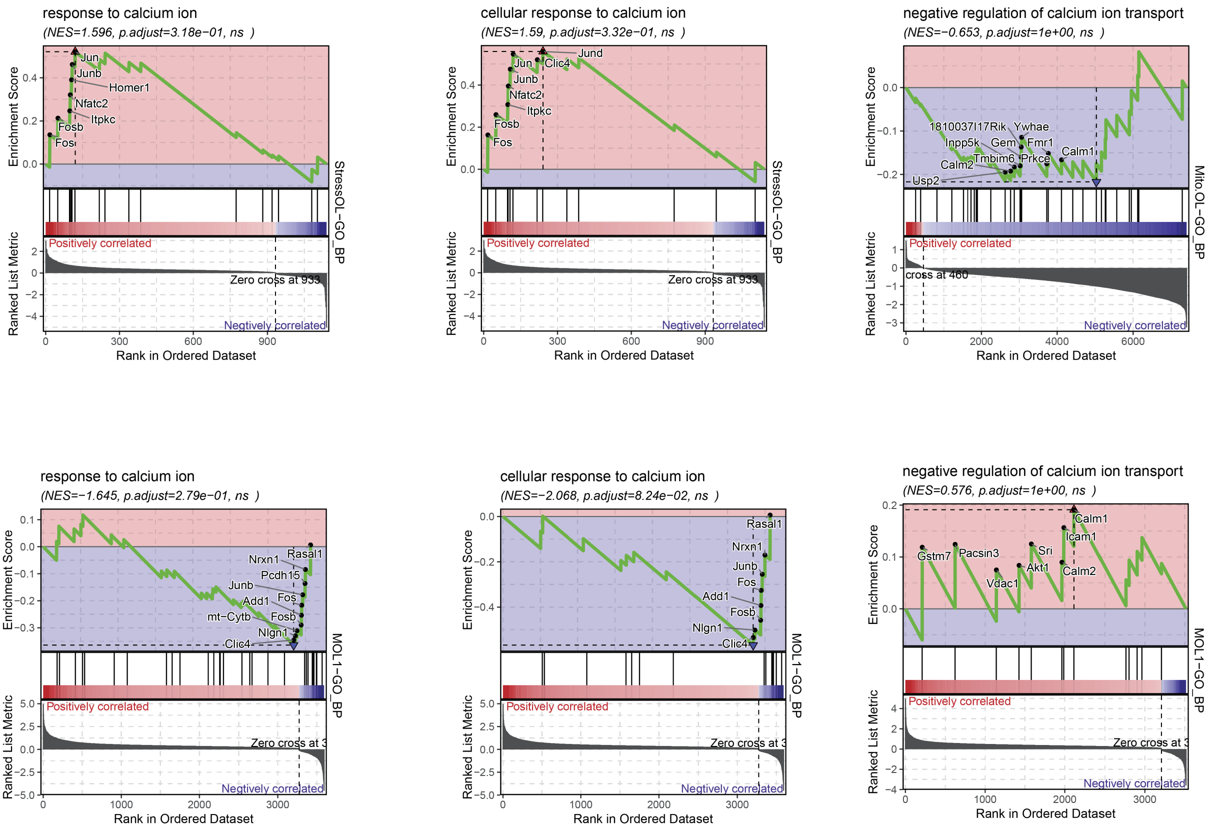

Supplementary Figure 11. GSEA plots for specific OL populations using calcium response gene sets

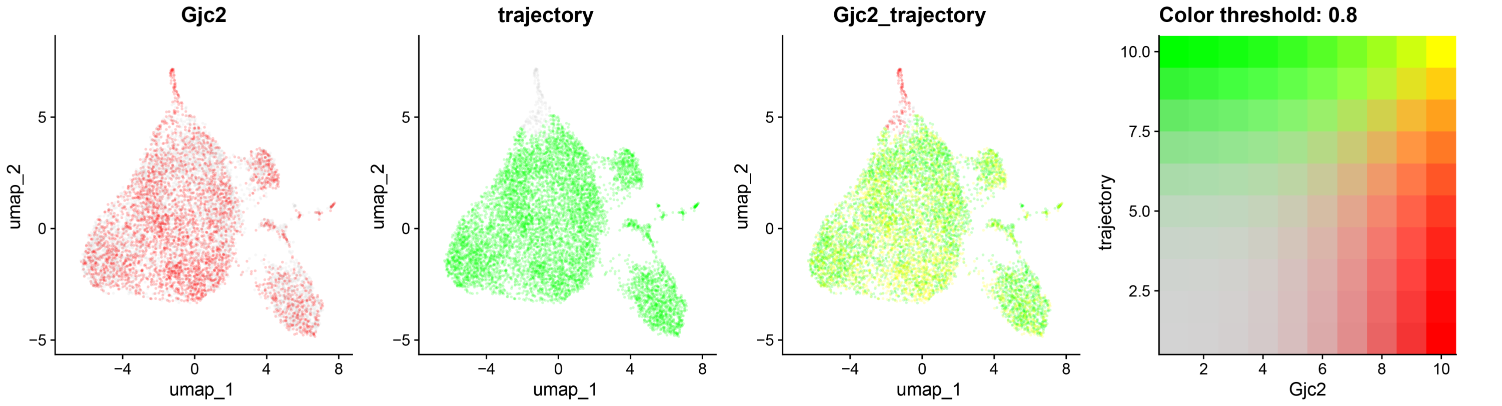

Supplementary Figure 12. Oligodendrocyte pseudotime umap overlaid with Gjc2 expression

This umap plot illustrates the oligodendrocyte pseudotime trajectory, overlaid with the expression levels of the Gjc2 gene. Corresponding color threshold panels are also shown.

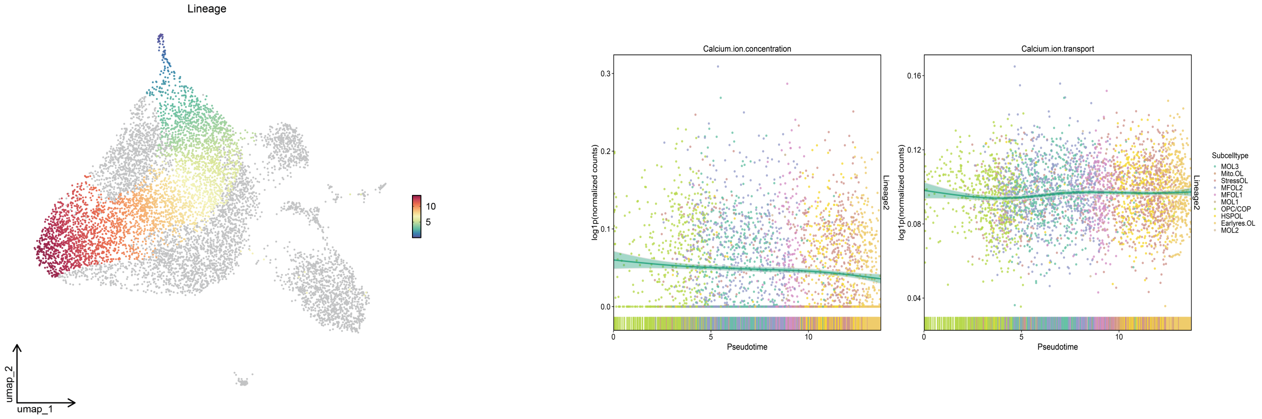

Supplementary Figure 13. Oligodendrocyte pseudotime trajectory and calcium dynamics

This figure includes a UMAP plot showing the oligodendrocyte pseudotime trajectory inferred using Slingshot. Additionally, a scatter plot depicts the changes in calcium ion concentration and calcium ion transport pathway activity along this pseudotime trajectory, with a fitted regression line.

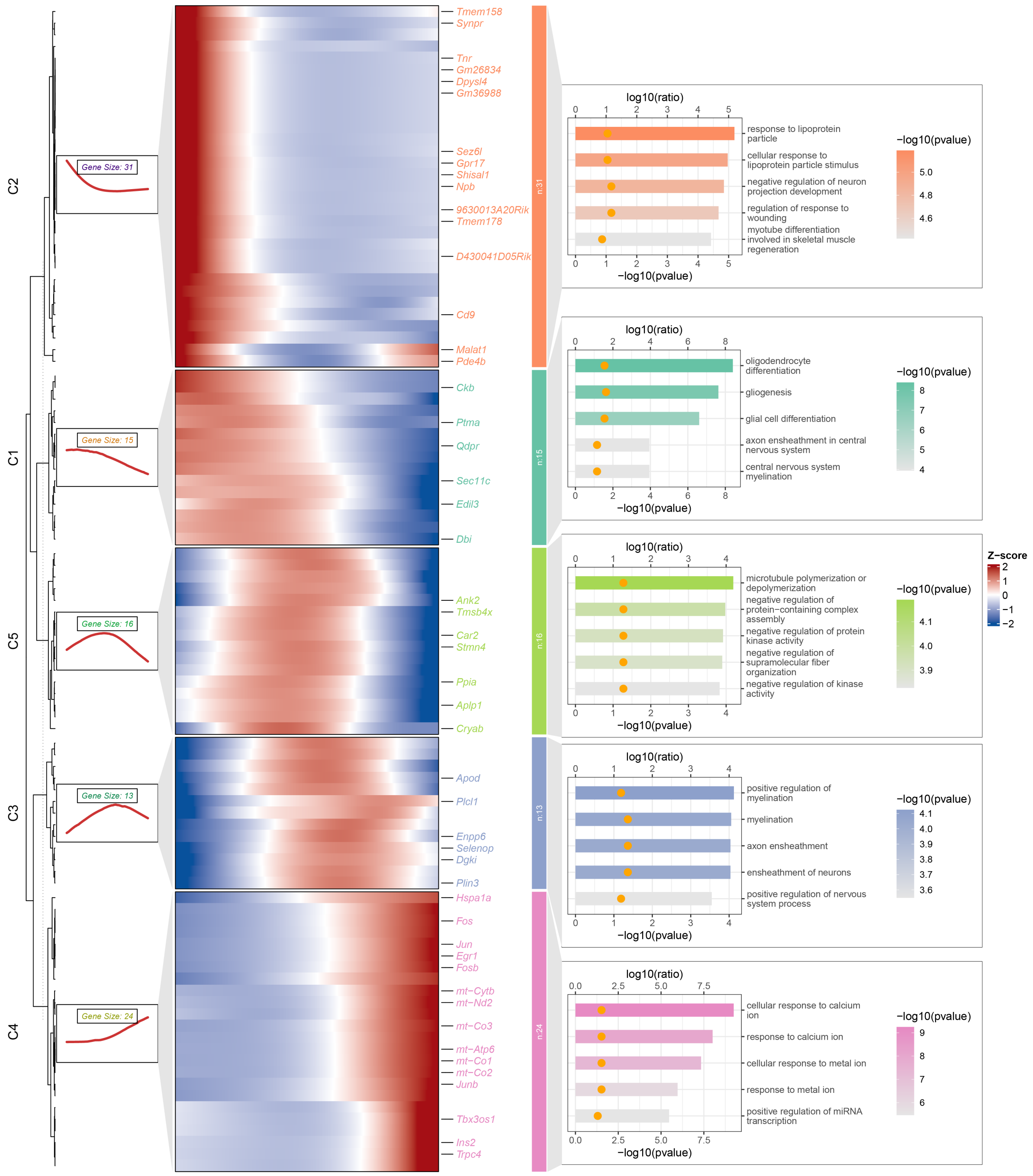

Supplementary Figure 14. Heatmap of pseudotime DEGs and their enrichment

This heatmap displays DEGs identified along the oligodendrocyte pseudotime trajectory, which was inferred using Monocle3. The top 50 genes are labeled. The left panels represent gene clusters and their expression trends over pseudotime, while the right panels show the top 5 GO_BP enrichments for each gene cluster.
